# COMP-prohibitin 2 interaction maintains mitochondrial homeostasis and controls smooth muscle cell identity

**DOI:** 10.1038/s41419-018-0703-x

**Published:** 2018-06-04

**Authors:** Yiting Jia, Meili Wang, Chenfeng Mao, Fang Yu, Yingbao Wang, Rui Xiao, Changtao Jiang, Lemin Zheng, Qingbo Xu, Ming Zheng, Yi Fu, Qinghua Hu, Wei Kong

**Affiliations:** 1 Department of Physiology and Pathophysiology, School of Basic Medical Sciences, Peking University Health Science Center, Beijing, China; 2 Key Laboratory of Molecular Cardiovascular Science, Ministry of Education, Beijing, China; 3 Department of Pathophysiology, School of Basic Medicine, Tongji Medical College, Huazhong University of Science and Technology, Wuhan, China; 4 The Institute of Cardiovascular Sciences and Institute of Systems Biomedicine, School of Basic Medical Sciences, and Key Laboratory of Molecular Cardiovascular Sciences of Ministry of Education, Peking University Health Science Center, Beijing, China; 5 Cardiovascular Division, Kings College London BHF Centre, London, UK

## Abstract

Vascular smooth muscle cells (VSMCs) are highly phenotypically plastic, and loss of the contractile phenotype in VSMCs has been recognized at the early onset of the pathology of a variety of vascular diseases. However, the endogenous regulatory mechanism to maintain contractile phenotype in VSMCs remains elusive. Moreover, little has been known about the role of the mitochondrial bioenergetics in terms of VSMC homeostasis. Herein, we asked if glycoprotein COMP (Cartilage oligomeric matrix protein) is involved in mitochondrial bioenergetics and therefore regulates VSMCs homeostasis. By using fluorescence assay, subcellular western blot and liquid chromatography tandem mass spectrometry analysis, we found that extracellular matrix protein COMP unexpectedly localized within mitochondria. Further mitochondrial transplantation revealed that both mitochondrial and non-mitochondrial COMP maintained VSMC identity. Moreover, microarray analysis revealed that COMP deficiency impaired mitochondrial oxidative phosphorylation in VSMCs. Further study confirmed that COMP deficiency caused mitochondrial oxidative phosphorylation dysfunction accompanied by morphological abnormality. Moreover, the interactome of mitochondrial COMP revealed that COMP interacted with prohibitin 2, and COMP–prohibitin 2 interaction maintained mitochondrial homeostasis. Additionally, disruption of COMP–prohibitin 2 interaction caused VSMC dedifferentiation in vitro and enhanced the neointima formation post rat carotid artery injury in vivo. In conclusion, COMP–prohibitin 2 interaction in mitochondria plays an important role in maintaining the contractile phenotype of VSMCs by regulating mitochondrial oxidative phosphorylation. Maintaining the homeostasis of mitochondrial respiration through COMP–prohibitin 2 interaction may shed light on prevention of vascular disease.

## Introduction

Vascular smooth muscle cells (VSMCs) are of high plasticity and can undergo reversible changes in phenotype in response to alterations in local environmental cues^1^. In mature blood vessels, VSMCs exhibit a “contractile” or “differentiated” phenotype characterized by the expression of contractile markers, including smooth muscle myosin heavy chain (SM-MHA), SM α-actin, SM-22 and calponin. However, under certain circumstances, VSMCs can undergo phenotype transition toward a non-contractile or dedifferentiated phenotype characterized by reduced expression of contractile markers and increased capacity for cell proliferation, migration, or secretion of various extracellular matrix (ECM) proteins and cytokines. Accumulating evidence has suggested that phenotypic transition of VSMCs occurs at the early onset of the pathology of several vascular diseases, including atherosclerosis, post-injury restenosis, vascular calcification and aortic aneurysms^2–5^. To date, compelling studies have identified the contribution of growth factors, mitogenic cytokines, reactive oxygen species, hypoxia, stretch and injury to cell dedifferentiation^6–9^. Recent studies have also revealed the importance of CArG-SRF-myocardin-dependent transcriptional mechanisms and epigenetic controls in the regulation of VSMC differentiation^10^. Nevertheless, how quiescent VSMCs maintain the differentiated state upon environmental cues is much less understood.

Mitochondria are the energy powerhouses for ATP production and are particularly abundant in muscle cells, including human VSMCs^11^. Until recently, little has been known about the role of the bioenergetics of VSMC mitochondria in terms of vascular function and disease. The bioenergetics of human VSMCs via mitochondrial metabolism mainly relies on oxygen-consuming mitochondrial oxidative phosphorylation (OXPHOS) due to high efficiency of ATP production, whereas glycolysis is also involved^12,13^. It has become increasingly evident that under certain disease conditions, there is damage to the mitochondria that limits their ability to generate ATP via oxidative phosphorylation, leading to mitochondrial dysfunction. Subsequently, mitochondrial dysfunction causes the overproduction of reactive oxygen species (ROS), mitochondrial DNA damage, aberrant mitochondrial dynamics and disturbed calcium homeostasis, ultimately contributing to cardiovascular diseases such as atherosclerosis^14–17^. A few studies have shown that during aortic VSMC dedifferentiation induced by serum or PDGF-BB, mitochondria undergo metabolic reprogramming and decreased ATP production^18^. Moreover, complex I dysfunction underlies the oxidative phosphorylation-to-glycolysis switch in pulmonary hypertensive smooth muscle cells^19^. However, the definitive role of mitochondrial respiration in the VSMC phenotypic transition and the underlying mechanism have been little studied.

Cartilage oligomeric matrix protein (COMP), a 524-kDa pentameric noncollagenous glycoprotein, is an extracellular matrix protein found in both the musculoskeletal and cardiovascular systems. Our recent studies have demonstrated that COMP plays key roles in the maintenance of VSMC homeostasis. COMP maintains the contractile phenotype of VSMCs via integrin α7β1 and prevents osteochondrogenic transdifferentiation of VSMCs by directly binding to BMP-2, which inhibits post-injury neointima formation and vascular calcification respectively^20,21^. We recently also demonstrated that COMP negatively regulates atherosclerosis and lesional calcification formation via direct interaction with integrin β3^22^. In the current study, we identified an unexpected intracellular localization of COMP and a role of the COMP–prohibitin 2 interaction in the maintenance of mitochondrial homeostasis and the regulation of VSMC phenotype switching. The prohibitins are highly conserved proteins in eukaryotic cells that are present in multiple cellular compartments including mitochondrion, cytoplasm and nucleus of a variety of cells^23,24^. In the mitochondrial inner membrane, prohibitin 1 and prohibitin 2 form a ring-like structure, thus maintains mitochondrial genome stability, mitochondrial dynamics and promotes mitochondrial respiratory chain complex assembling^25–28^. So far, little is known about the role of prohibitin 2 in VSMC homeostasis.

## Materials and Methods

### Materials

Antibodies against COMP, TGN46, ERp5, SM22, calponin, ATP synthase subunit β, β-tubulin, α-actin and β-actin used for the western blot analysis, were purchased from Abcam (Cambridge, UK). Antibodies against GAPDH, His were obtained from Cell Signaling Technology (Boston, MA, USA). Antibodies against prohibitin 1, prohibitin 2 and eIF-5 were purchased from Santa Cruz Biotechnology (Santa Cruz, CA, USA). IRDye-conjugated secondary antibodies for western blotting were purchased from Rockland, Inc. (Gilbertsville, PA, USA). JC-1, Mito-Tracker and antibody against COMP used for confocal fluorescence microscopy were purchased from Thermo Fisher Scientific (Thermo, NY, USA). Mito stress kit and cell energy phenotype kit were purchased from Agilent Seahorse XF Technology (Seahorse, CA, USA). PGC-1α plasmid was kindly provided by Prof. Changtao Jiang from Peking university. Other reagents were obtained from Sigma-Aldrich (St. Louis, MO, USA) unless specified.

### Animal preparation

COMP^−/−^ mice in the C57BL/6J background were kindly provided by Professor Äke Oldberg (Department of Cell and Molecular Biology, University of Lund, Sweden)^29^. Eight-week-old male and female COMP^−/−^ mice and their wild-type (WT) littermates were used for VSMC isolation. All animal studies followed the guidelines of the Animal Care and Use Committee of Peking University.

### Isolation of the mitochondrial fraction

Mitochondria were isolated by sucrose density gradient centrifugation according to a well-established protocol^30^. All steps were performed at 0–4 °C. Briefly, cells (5 × 10^7^) were collected and washed twice with cold PBS. The cells were subsequently washed with 10 to 20 ml of TD buffer (134 mM NaCl, 5 mM KCl, 0.7 mM Na_2_HPO_4_, and 2.5 mM Tris, pH 7.5). Following centrifugation, the pellet was resuspended in 10 ml of MgRSB buffer (10 mM NaCl, 1.5 mM MgCl_2_, and 10 mM Tris, pH 7.5) and incubated for 10 min to swell. Swollen cells were collected in a glass homogenizer with 40 strokes, when ~70% of the cells were disrupted. To stabilize the mitochondria, 2.5× mannitol-sucrose buffer was added to a final concentration (210 mM mannitol, 70 mM sucrose, 5 mM Tris, and 5 mM EDTA, pH 7.5). Following the removal of the nuclei (3000 r.p.m., 10 min, 2 times), the mitochondria were pelleted from this supernatant at 15,000 r.p.m. for 20 min. Following resuspension in 5 ml of mannitol-sucrose buffer, the pellet was laid over a discontinuous sucrose gradient consisting of 15 ml of 1.0 M sucrose, 5 mM EDTA, and 10 mM Tris, pH 7.5, on top of 15 ml of 1.5 M sucrose, 5 mM EDTA, and 10 mM Tris, pH 7.5, and then was centrifuged at 4 °C for 30 min at 22,000 rpm. The components at the 1.0–1.5 M sucrose interface were collected as mitochondria.

### Western blotting

Western blotting was performed as previously described^31^. Cells and mouse tissue extracts that contained equal amounts of total protein were resolved by 10 or 12% SDS-PAGE and were subsequently transferred onto nitrocellulose or PVDF membranes. The membranes were blocked with 5% milk in TBST, followed by incubation with primary antibody at 4 °C overnight. Following 1 h of incubation with IRDye-conjugated secondary antibodies (Rockland, Inc., Gilbertsville, PA, USA), the membranes were analyzed using an Odyssey infrared imaging system (LI-COR Biosciences, Lincoln, NE, USA) to detect the immunofluorescence signal.

### Mitochondrial subfractionation

Approximately 1 mg of mitochondria was sufficient for subfractionation as described^32^. All steps were performed at 0–4 °C. Briefly, mitochondria were suspended with 40 µl of T10E20 buffer (10 mM Tris-Cl, and 1 mM EDTA, pH 7.6) and were subsequently incubated with digitonin (0.1 mg digitonin/mg of mitochondrial protein). Three volumes of 250 mM sucrose in T10E20 buffer were added. Following centrifugation at 10,000 × *g* for 15 min, the inner mitochondrial membrane and matrix of the mitochondria were precipitated, whereas the outer membrane and intermembrane components were in the supernatant.

### N-terminal sequencing

The mitochondrial and residual cytoplasmic fractions were isolated from HEK293A cells transfected with the full-length COMP plasmid as previously described, and the lysates were incubated with anti-COMP antibody. The precipitated proteins were denatured with 2× SDS sample buffer at 95 °C for 5 min and were subsequently loaded onto 8% gradient gels. Following coomassie brilliant blue staining, the protein bands present exclusively (110 kDa) were excised respectively. The N-terminus of each protein in the mitochondrial and non-mitochondrial cytoplasmic bands was labeled with a dimethyl moiety before mass spectrometric analysis, and each N-terminus of the trypsin post-digestive peptide fragment was sequenced for analysis.

### Mitochondria transplantation

Mitochondria were transplanted as previously described^33,34^. To remove endogenous mitochondria, VSMCs at 80% confluence in culture were either maintained in DMEM as control or treated in DMEM supplemented with 110 µg/ml sodium pyruvate, 50 µg/ml uridine, and different concentrations of ethidium bromide (50, 100, or 200 ng/ml), respectively. After 2 weeks, mtDNA copy number was detected to verify removal efficiency. Then the VSMCs were incubated with 2.35 × 10^8^/ml GFP-labeled exogenous mitochondria prepared from scrambled siRNA or COMP siRNA-transfected VSMCs at 37 °C for 24 h. The transplantation of exogenous mitochondria was evaluated and quantified by live cell imaging using mitochondria-targeted GFP as tracer. The oxygen consumption rate measurement was performed to detect the function of transplanted mitochondria.

### Microarray analysis

Primary aortic VSMCs were isolated from WT and COMP^−/−^ mice, and the mRNA was extracted from 5 × 10^6^ cells isolated from 6 mice per sample using Trizol reagent (Invitrogen, CA, USA). The RNA quantity and quality were measured using a NanoDrop ND-1000 spectrophotometer, and the RNA integrity was assessed using standard denaturing agarose gel electrophoresis. The microarray experiments were performed using Kang Chen Bio-technology Corp (Shanghai, China) according to the standard protocol in three independent repeats. A Mouse DNA Array (Roche NimbleGen, Madison, WI, USA) was used to compare the gene expression between WT and COMP^−/−^ VSMCs.

Briefly, the total RNA from each sample was used for labeling and array hybridization via the following steps: (1) reverse transcription using Invitrogen SuperScript ds-cDNA synthesis kit; (2) ds-cDNA labeling using the NimbleGen one-color DNA labeling kit; (3) array hybridization using the NimbleGen Hybridization System, followed by washing with the NimbleGen wash buffer kit; and (4) array scanning using the Axon GenePix 4000B microarray scanner (Molecular Devices Corporation). Scanned images (TIFFs) were subsequently imported into NimbleScan software (Version 2.5) for grid alignment and expression data analysis. The expression data were normalized using quantile normalization and the Robust Multichip Average (RMA) algorithm included in the NimbleScan software. All gene level files were imported into Agilent GeneSpring GX software (Version 11.5.1) for further analysis. Genes that were expressed at significantly different levels between the groups were identified through volcano plot filtering. Briefly, the *p* value of *t* test between the two groups were corrected by false discovery rate (FDR). Then a volcano plot was introduced to display log-fold-change against −log10(*p* value) from the *t* test. The cutoff of *p* value is 0.01. Differentially expressed genes were identified through fold change filtering (>2.0; <0.5). Pathway Analysis (KEGG) and GO analysis were used to analyze the functions of the differentially expressed genes.

### Measurement of the mitochondrial oxygen consumption rate (OCR)

The OCRs were measured as previously described using an XF24 Extracellular Flux Analyzer (Seahorse Biosciences)^35^. Rat VSMCs were seeded in XF24 culture plates at a density of 20,000 cells per well overnight and were cultured with DMEM that contained 10% FBS. The direct measurement was recorded as the baseline OCR, followed by the addition of oligomycin (2 μM) to measure the ATP-linked OCR, and the oxidative phosphorylation uncoupler FCCP (2 μM) to indicate the maximal respiration. Finally, rotenone (1 μM) and antimycin A (1 μM) were injected to determine non-mitochondrial respiration. The operations followed the manufacturer’s protocol. The OCR was normalized by the amount in 100 μg of cellular protein in each well.

### Recombinant adenovirus construction and infection

An adenovirus for full-length mouse COMP (Ad-COMP; NM_016685.2) was constructed and amplified according to the manufacturer’s protocol (BD Biosciences). An adenovirus that carried GFP (Ad-GFP) was used as a negative control. Cells cultured at ~80% confluence were infected with recombinant adenovirus (50 multiplicity of infection) for 48 h.

### Plasmid construction and transfection

The cDNA fragment that encoded full-length rat prohibitin 2 (NCBI Reference sequence: NM_001013035.1) was cloned into the BamHI/XbaI sites of the pcDNA3.1 plasmid. The full-length COMP was subcloned into an C-terminal RFP vector for expression in primary mouse vascular smooth muscle cells. The full-length COMP coding sequence and fragments that encoded the 4 functional domains of mouse COMP (i.e., the N-terminus [N; aa 1–83], epidermal growth factor (EGF) repeat domain [EGF; aa 84–261], type III repeat domain [type III; aa 266–520], and C-terminus [C; aa 521–755]) were subcloned into an N-terminal FLAG vector for expression in COS-7 cells. The full-length prohibitin 2 coding sequence and fragments that encoded the 3 functional domains of mouse prohibitin 2 (i.e., the N-terminus [N; aa 1–39], prohibitin (PHB) domain [PHB; aa 34–264], and C-terminus [C; aa 201–299]) were subcloned into an N-terminal 6 × His vector for expression in HEK293A cells. In vitro plasmid transfection was performed using jetPEI (Polyplus-Transfection SA, Illkrich, France). The transfection procedures followed the manufacturers’ instructions and all the primers used for subcloning are shown in Supplemental Table III.

### Rat carotid artery balloon injury

Male Sprague–Dawley rats (210–230 g) were used for the carotid artery injury model. All studies followed the guidelines of the Animal Care and Use Committee of Peking University. Briefly, rats were anesthetized by intraperitoneal injection of chloralhydrate (300 mg/kg). A 1.5-mm diameter balloon catheter (Medtronic, Minneapolis, Minn) was introduced through the left external carotid artery and distended then pulled back with constant rotation. This procedure was repeated for 5 times. Contralateral carotid arteries served as sham controls.

### Recombinant adenovirus construction and infection in mice

The prohibitin 2 dominant-negative fragment (prohibitin 2-DN, aa201–299) was subcloned into an adenovirus vector pAdTrack-CMV. Subsequently, the recombinant adenovirus plasmids pDC316-mCMV-prohibitin 2-DN were transfected into HEK293A cells to yield the final expression clone Ad-prohibitin 2-DN. Ad-prohibitin 2-DN was amplified by infecting HEK293A cells and purified by PD-10 Sephadex (GE healthcare, Marlborough) precipitation. An adenovirus vector carrying green fluorescence protein (Ad-GFP) was applied as negative control. For in vivo studies, 1 × 10^10^ pfu of adenovirus dissolved in 30% pluronic gel solution were perivascularly delivered to the carotid arteries post balloon injury as described previously^21^.

### Statistical analysis

All the results of the experimental studies are expressed as the mean ± SD. Statistical analysis was performed using GraphPad prism 6.0 software (GraphPad Software, San Diego, California, USA). Whether data are normally distributed was first evaluated. Then Brown-Forsythe test was performed for checking similar variances among normally distributed data followed by Student’s *t* test for two-group comparisons and ANOVA for more than two-group comparisons if evaluation of similar variances was passed. Nonparametric tests were used where data were not normally distributed. In all cases, statistical significance was concluded where the two-tailed probability was less than 0.05. Briefly, paired two-tailed Student’s *t* test was applied for the comparisons of the protein expression and mitochondrial membrane potential between the VSMCs transfected with scrambled siRNA and COMP siRNA, whereas unpaired two-tailed Student’s *t*-test was applied for the comparisons of the percentages of ATP production and amount of mtDNA between the VSMCs transfected with scrambled siRNA and COMP siRNA, as well as the comparisons of the mRNA levels between the WT and COMP^−/−^ VSMCs, the comparation of neointima area, neointima/media ratio, circumference of EEL, the media area between Ad-GFP and Ad-prohibitin 2-DN infected rat carotid arteries. The comparisons of the protein expression among more than 2 groups by western blot were analyzed using one-way ANOVA followed by the Student–Newman–Keuls test for post-hoc comparison. The comparisons of the OCRs between the VSMCs treated with scrambled siRNA or COMP siRNA and control or COMP-deficient mitochondria as well as vector or prohibitin 2-DN plasmid were analyzed by unpaired two-tailed Student’s *t* test. Specific statistical analysis used in each experiment was described in detail in figure legends.

## Results

### COMP intracellularly localized in mitochondria

**Fig. 1:**
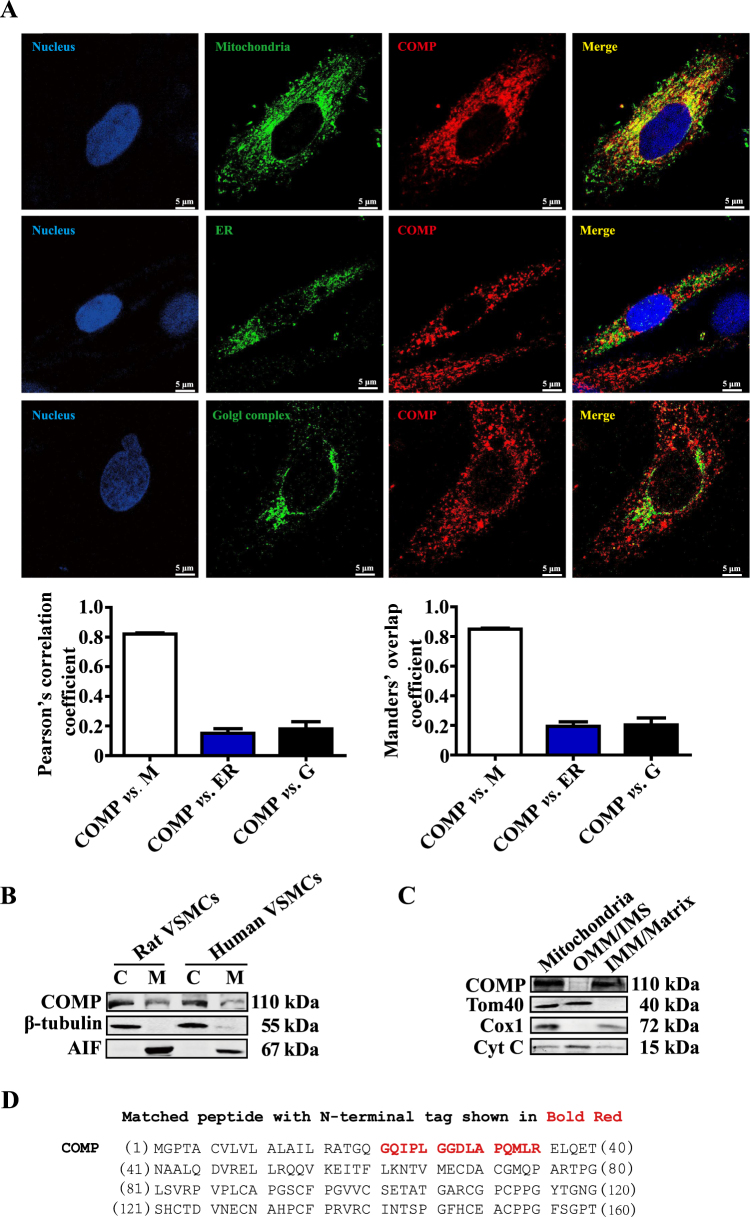

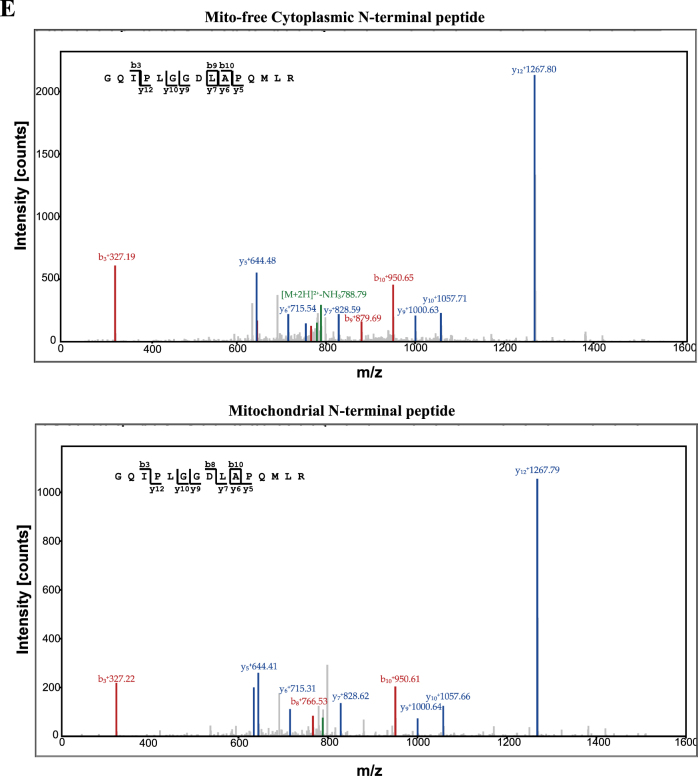
COMP is intracellularly localized in mitochondria. **a** Subcellular localization of COMP as demonstrated by confocal fluorescence microscopy. Mito-Tracker, TGN46 and ERp5 were used to indicate the mitochondria, Golgi complex and endoplasmic reticulum, respectively. Colocalization was evaluated on basis of the Pearson’s correlation coefficient and Manders’ overlap coefficient from 20–30 cells in 4 independent studies\. Scale bar = 5 μm. **b** Cell fractionation prior to the western blot analysis of COMP expression. Tubulin and AIF were used as cytoplasmic and mitochondrial markers, respectively. **c** Mitochondria isolated from rat VSMCs were subjected to subfractionation prior to the identification of COMP by western blot. Tom40 and Cox1 were used as markers for the mitochondrial outer and inner membranes, respectively. Cytochrome c is a dynamic component of mitochondria and is present in both the inner membrane and intermembrane space. **d** Sequence of mitochondrial and non-mitochondrial cytoplasmic COMP. The potential common N-terminus of mitochondrial COMP and non-mitochondrial cytoplasmic COMP is in bold red. **e** The MS/MS spectrum of the peptide (GQIPLGGDLAPQMLR) that matches the N-terminal sequence of non-mitochondrial cytoplasmic and mitochondrial COMP. M mitochondria, ER endoplasmic reticulum, G golgi complex, C cytoplasm, M mitochondria, OMM outer mitochondrial membrane, IMS intermembrane space, IMM inner mitochondrial membrane, Matrix mitochondrial matrix

We previously showed that the ECM protein COMP is essential for maintaining the contractile phenotype of VSMCs, and siRNA-mediated silencing of COMP causes VSMC dedifferentiation^20^. Whether this is true in COMP^−/−^ VSMCs was not explored. In the current study, we isolated primary VSMCs from COMP^−^^/−^ and wild-type mice. COMP^−/−^ VSMCs exhibited the decreased levels of contractile proteins, including α-actin, calponin and SM22 as well as morphological alteration to polygonal-shaped cells, which was rescued by the ectopic expression of COMP with red fluorescence protein labeled COMP (COMP-RFP) plasmid (Supplemental Figure IA-B). Intriguingly, we found an unexpected intracellular distribution of COMP-RFP in COMP^−^^/−^ VSMCs (Supplemental Figure IC). To exclude the non-specific interference of COMP-RFP on COMP distribution, we subsequently performed dual immunofluorescence analysis to detect endogenous COMP expression in rat VSMCs. Surprisingly, COMP intracellularly localized and mainly resided in the mitochondria rather than the endoplasmic reticulum and Golgi complex, as evidenced by co-immunostaining of COMP with Mito-Tracker but not ERp5 or TGN46 in VSMCs (Fig. 1a). Moreover, we isolated mitochondrial and residual cytoplasmic fractions from rat and human VSMCs. Western blot analysis indicated that COMP existed in both the mitochondria and cytoplasm (Fig. 1b). Mitochondria have the following four compartments: the outer mitochondrial membrane (OMM), the intermembrane space (IMS), the inner mitochondrial membrane (IMM), and the mitochondrial matrix^36^. Upon further investigation of submitochondrial localization, we found that COMP preferentially localized in the IMM/matrix but was less abundant in the OMM/IMS (Fig. 1c). Together, the extracellular matrix protein COMP also localizes intracellularly in the mitochondria of VSMCs.

Most nuclear DNA coded mitochondrial proteins have amphiphilic N-terminal mitochondrial targeting sequence, which will be cut after translocated into mitochondria^37^. By using Mitofate (http://mitf.cbrc.jp/MitoFates/cgi-bin/top.cgi) software, the N-terminus (residues 12–23) of COMP is predicted to be responsible for mitochondrial targeting (data not shown). To investigate the definite sequence of mitochondrial COMP and cytoplasmic COMP, we subcloned the full-length of COMP and transfected it into HEK293A cells. Mitochondrial and mitochondria-free cytoplasmic fractions were isolated followed by immunoprecipitation. After Coomassie brilliant blue staining, the protein band of COMP (between 100 and 130 kDa) in each fraction was excised, and the N-terminus was labeled with a dimethyl moiety, followed by subjection to LC-MS/MS analysis (Supplemental Figure ID). Mascot reports revealed the same pre-labeled N-terminal peptide of COMP (15 aa, MS/MS spectrum shown in Fig. 1e) in mitochondria-free cytoplasmic and mitochondrial fractions. The N-termini of mitochondria-free cytoplasmic and mitochondrial COMP are both mapped from Gly21, suggesting that cytoplasmic and mitochondrial COMP share the same N-terminal signal peptide (aa 1–20) (Fig. 1d).

### Mitochondrial COMP controls the contractile phenotype of VSMCs

**Fig. 2:**
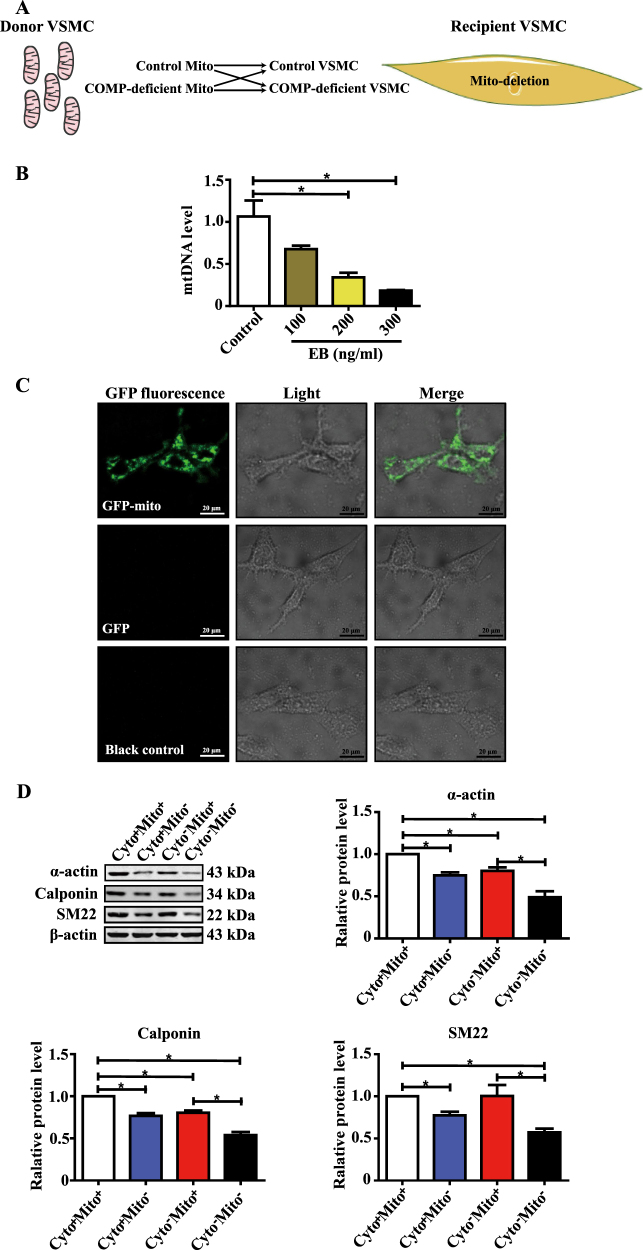
Mitochondrial COMP maintains the contractile phenotype of VSMCs. **a** Systematic view of mitochondrial transplantation. Mito, mitochondria. **b** Mitochondrial DNA copy number of VSMCs treated with ethidium bromide for two weeks. The data was analyzed using one-way ANOVA followed by the Student–Newman–Keuls test for post-hoc comparison and presented as the means ± SD of five independent experiments. *P < 0.05. **c** Live-cell confocal imaging of VSMCs after incubation with GFP-labeled mitochondria prepared from VSMCs (upper), purified GFP (middle) or vehicle (lower). Scale bar = 20 μm. **d** Scrambled siRNA-transfected and COMP siRNA-transfected VSMCs transplanted with scrambled or COMP siRNA-transfected mitochondria, underwent western blot analysis and quantification of the protein levels of α-actin, calponin and SM22. The data were analyzed using one-way ANOVA followed by the Student–Newman–Keuls test for post-hoc comparison and presented as the means ± SD of six independent experiments. Cyto^+^Mito^+^ indicates control VSMCs transplanted with control mitochondria, Cyto^+^Mito^-^ indicates control VSMCs transplanted with COMP-deficient mitochondria, Cyto^-^Mito^+^ indicates COMP-deficient VSMCs transplanted with control mitochondria, Cyto^-^Mito^-^ indicates COMP-deficient VSMCs transplanted with COMP-deficient mitochondria. *P < 0.05

To further investigate whether mitochondrial or non-mitochondrial COMP is responsible for VSMC identity, we performed mitochondrial transplantation as described previously^33,34^. The mitochondria were isolated from scrambled or COMP- silenced donor VSMCs and then transplanted into recipient mitochondria-deleted VSMCs treated with scrambled or COMP siRNA respectively (Fig. 2a). The efficiency of mitochondrial deletion after ethidium bromide stimulation and mitochondria transplantation was examined by mtDNA level and live-cell confocal imaging respectively (Fig. 2b,c). Oxygen consumption rate of post-transplanted VSMCs measured using the Seahorse XF24 flux analyzer was performed to determine the function of transplanted mitochondria (data not shown). Both mitochondrial-deficiency and non-mitochondrial-deficiency of COMP contributed to VSMC dedifferentiation, whereas dual deficiency of COMP caused more significant alteration. Even though COMP in the mitochondrial fraction was minor compared to the mito-free cytosolic fraction (Fig. 1b and Supplemental Figure ID), our data suggested that mitochondria-resident COMP also contributed to VSMC contractile phenotype maintenance (Fig. 2d).

### COMP^−/−^ VSMCs exhibit dysfunction of mitochondrial oxidative phosphorylation

**Fig. 3:**
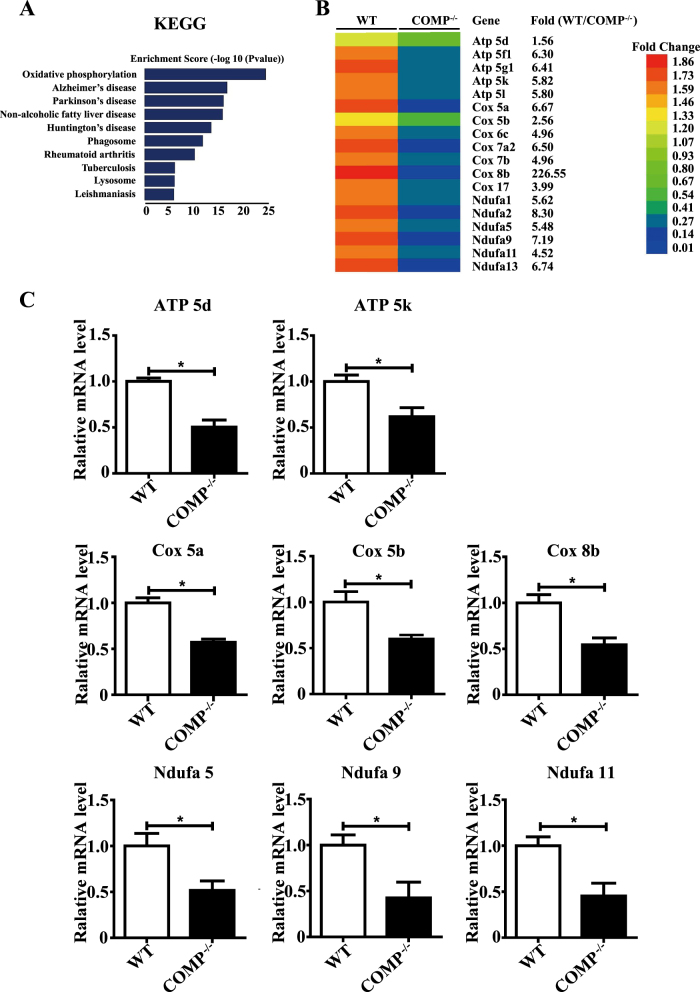

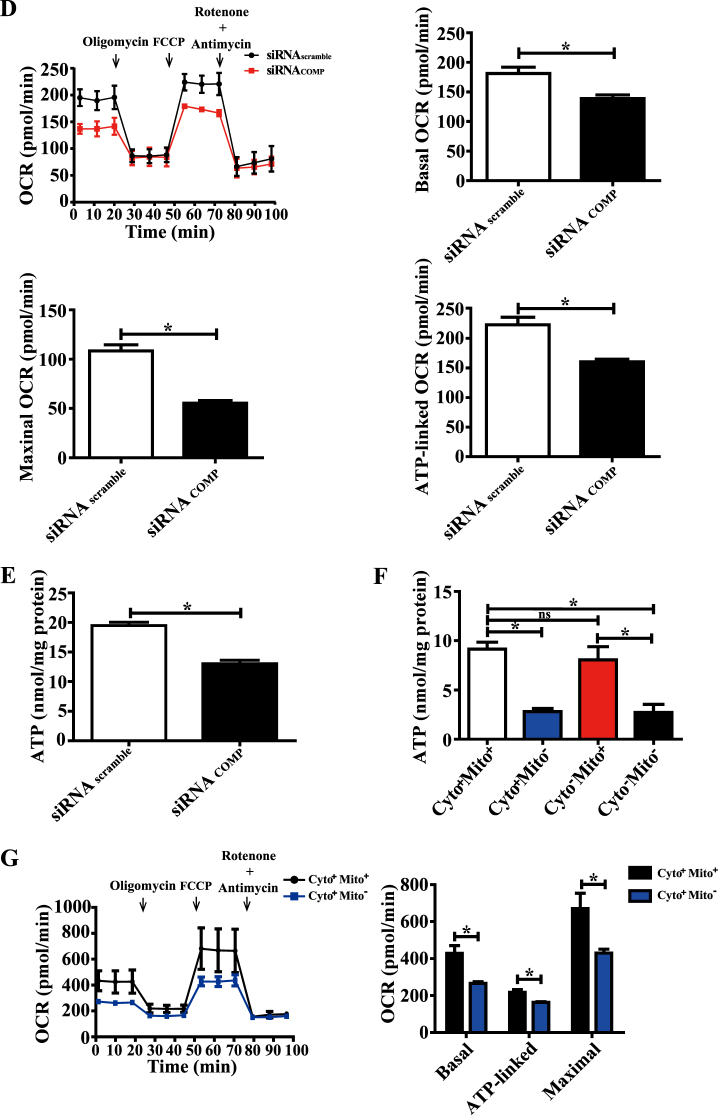
COMP^−/−^ VSMCs exhibit mitochondrial oxidative phosphorylation dysfunction. **a** Microarray analysis of the KEGG pathway downregulated by COMP deficiency. **b** Heatmap of representative genes involved in oxidative phosphorylation. **c** RT-qPCR validation of the genes related to oxidative phosphorylation downregulated by COMP deficiency in VSMCs. The data was analyzed using unpaired two-tailed Student’s *t* test and presented as the means ± SD of three independent experiments. *P < 0.05. **d** Traces of mitochondrial oxygen consumption rates of rat VSMCs measured using the Seahorse XF24 flux analyzer, with sequential injections of mitochondrial effectors at time points indicated by the downward arrows. The data were analyzed using unpaired two-tailed Student’s *t* test and presented as the means ± SD of four independent experiments. *P < 0.05. **e** Intracellular ATP production of rat VSMCs. The data were analyzed using unpaired two-tailed Student’s *t* test and presented as the means ± SD of four independent experiments. *P < 0.05. **f** Intracellular ATP production of scrambled siRNA-transfected and COMP siRNA-transfected VSMCs transplanted with scrambled or COMP siRNA-transfected mitochondria. The data were analyzed using one-way ANOVA followed by the Student–Newman–Keuls test for post-hoc comparison and presented as the means ± SD of four independent experiments in duplicate. *P < 0.05. **g** Traces and quantification of the mitochondrial oxygen consumption rates of scrambled siRNA-transfected VSMCs transplanted with scrambled or COMP siRNA-transfected mitochondria. The data were analyzed using unpaired two-tailed Student’s *t* test and presented as the means ± SD of six independent experiments. *P < 0.05

Previously, we have shown that extracellular COMP binds to integrin α7β1 to maintain the contractile phenotype of VSMCs^20^. To further assess how intracellular COMP regulates this process, we performed gene-expression microarray analysis between primary WT and COMP^−/−^ VSMCs. Overall, 7237 genes were upregulated, whereas 6389 genes were downregulated by COMP deficiency. GO and KEGG analysis revealed that those genes regulated by COMP deficiency were involved in multiple pathways, including metabolic processes, cell binding and oxidative phosphorylation (Supplemental Figure II). Intriguingly, among these pathways, the genes related to mitochondrial oxidative phosphorylation were most significantly downregulated in response to COMP deficiency (Fig. 3a, b). To validate the array data, we performed RT-qPCR analysis in primary WT and COMP^−/−^ VSMCs regarding the genes related to the activity of mitochondrial respiratory chain complexes and oxidative phosphorylation (ATP 5d, ATP 5k, Cox 5a, Cox 5b, Cox 8b, Ndufa 5, Ndufa 9 and Ndufa 11). Similar results were obtained (Fig. 3c). To determine whether COMP deficiency truly affects mitochondrial oxidative phosphorylation, we subsequently measured the oxygen consumption rate (OCR) between the VSMCs transfected with scrambled or COMP siRNA using an XF24 flux analyzer. The measurement was performed by sequential injection of the ATP synthase inhibitor oligomycin, uncoupler FCCP, and the combination of the electron transport chain inhibitors rotenone and antimycin A. Both the basal mitochondrial respiration and the maximal respiratory capacity were attenuated in the COMP-silenced VSMCs compared with the scrambled siRNA-transfected VSMCs. Moreover, oligomycin caused less reduction of the mitochondrial OCR in the COMP-silenced VSMCs than in the scrambled control cells, indicating that ATP synthesis-linked OCR was attenuated by COMP deficiency (Fig. 3d). Consistently, ATP production was significantly decreased in the COMP-silenced VSMCs compared with the control VSMCs (Fig. 3e). We further investigated whether deficiency of mitochondrial or non-mitochondrial cytoplasmic COMP is responsible for ATP production. After mitochondria transplantation, we found that deficiency of mitochondrial-resident COMP, but not non-mitochondrial COMP, was responsible for ATP production (Fig. 3f, g). In accordance, COMP deficiency in mitochondria caused decreased mitochondrial respiration. These findings indicate that COMP deficiency disrupted mitochondrial bioenergetics in VSMCs.

### COMP deficiency causes mitochondrial dysfunction

**Fig. 4:**
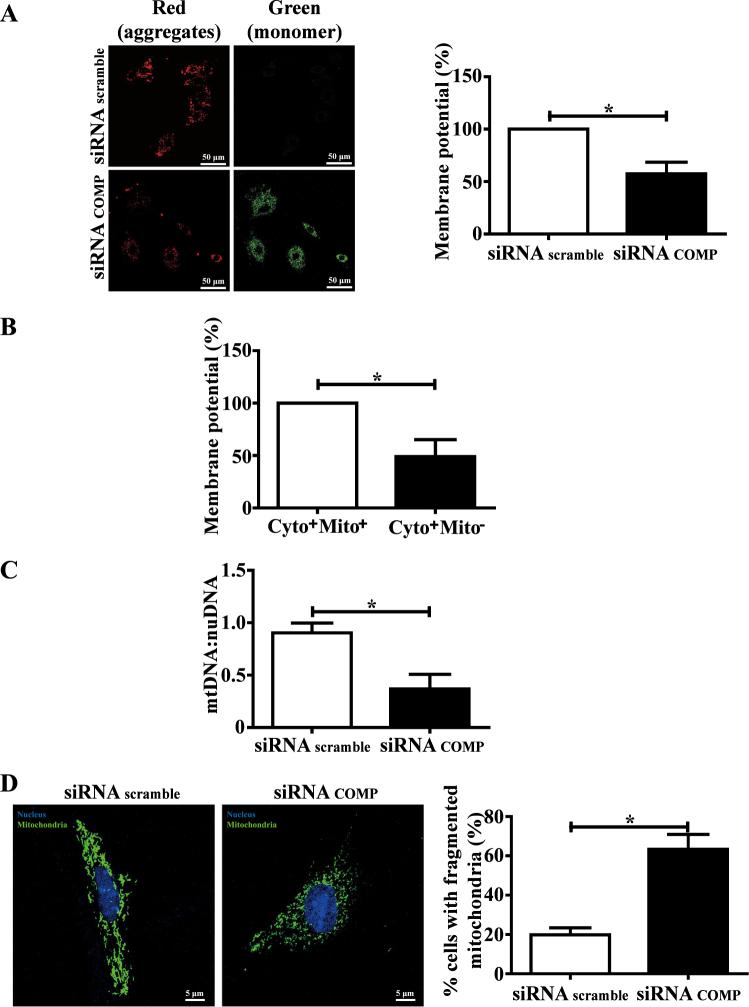
COMP deficiency leads to mitochondrial dysfunction. **a** JC-1 staining showing diminished membrane potential as confirmed by confocal fluorescence microscopy (left, Scale bar = 50 μm) and flow cytometry (right), the data were analyzed using paired two-tailed Student’s *t* test and presented as the means ± SD of three independent experiments. *P < 0.05. **b** Flow cytometry analysis of JC-1 staining in scrambled siRNA-transfected VSMCs transplanted with scrambled or COMP siRNA-transfected mitochondria. The data were analyzed using paired two-tailed Student’s *t* test and presented as the means ± SD of three independent experiments. *P < 0.05. **c** The mtDNA content was quantified by qPCR. The data were analyzed using unpaired two-tailed Student’s *t* test and presented as the means ± SD of three independent experiments. *P < 0.05. **d** Mitochondrial morphology was indicated in a rat VSMC using Mito-Tracker Green (left, scale bar = 5 μm). Mitochondrial length was measured using Image pro plus 6.0 software (Media Cybernetics, MD, USA), the data were analyzed using unpaired two-tailed Student’s *t* test and presented as the means ± SD of four independent experiments. *P < 0.05

Impaired oxidative phosphorylation has been proposed as a primary factor initiating mitochondrial dysfunction, which is characterized by decreased mitochondrial membrane potential (ΔΨm), reduced mitochondrial DNA (mtDNA) number, reduced autophagic flux and mitochondrial morphological changes. We firstly measured the mitochondrial membrane potential (△Ψm) using JC-1 dye staining. COMP silencing consistently decreased the intracellular red fluorescence signaling but enhanced green fluorescence signaling in the VSMCs, suggesting that △Ψm was significantly attenuated in the COMP-silenced VSMCs (Fig. 4a). Additionally, mitochondrial-resident COMP knockdown attenuated the mitochondrial membrane potential (Fig. 4b). Moreover, the relative content of mitochondrial DNA (mtDNA) was significantly reduced in COMP-silenced VSMCs (Fig. 4c).

Mitochondria have been recognized as highly dynamic and constantly undergo fission (the production of short mitochondrial rods or spheres) and fusion (the promotion of a long, filamentous morphology of mitochondria), which maintains mitochondrial homeostasis^38^. Decreased fusion or increased fission results in mitochondrial fragmentation, an increase in the number of abnormal mitochondria and VSMC proliferation^39^. COMP-silenced VSMCs exhibited substantial mitochondrial fragmentation, as indicated by Mito-Tracker staining (Fig. 4d).

Together, the data showed that COMP deficiency impaired mitochondrial oxidative phosphorylation and caused mitochondrial dysfunction.

### Mitochondrial bioenergetics defects by COMP deficiency contributes to VSMC dedifferentiation

**Fig. 5:**
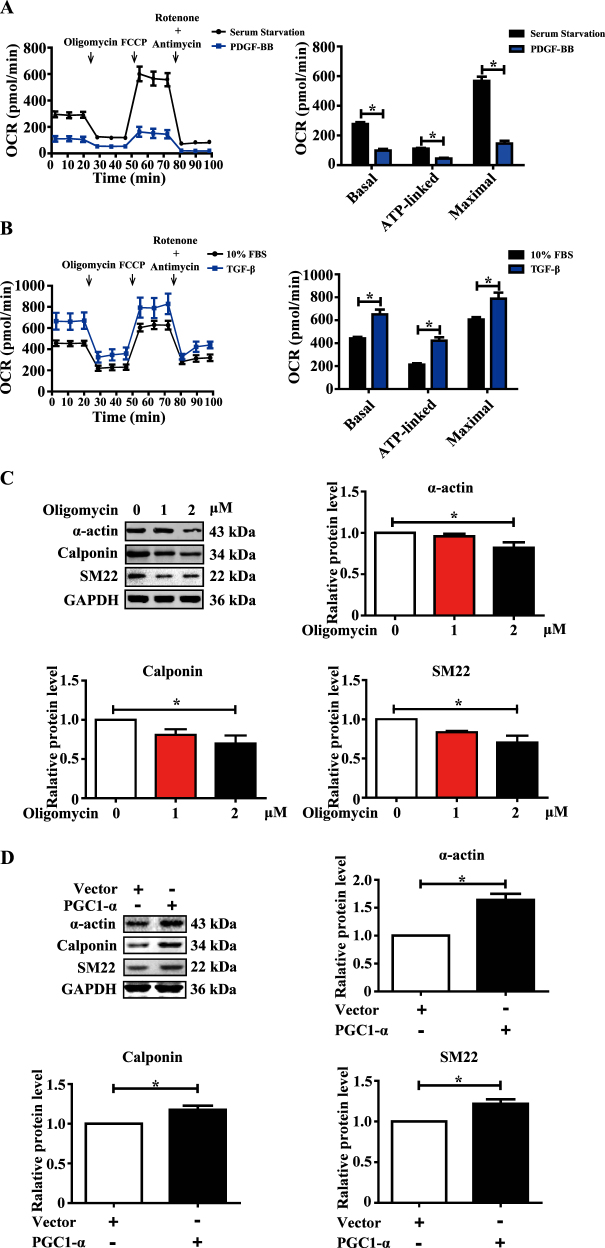

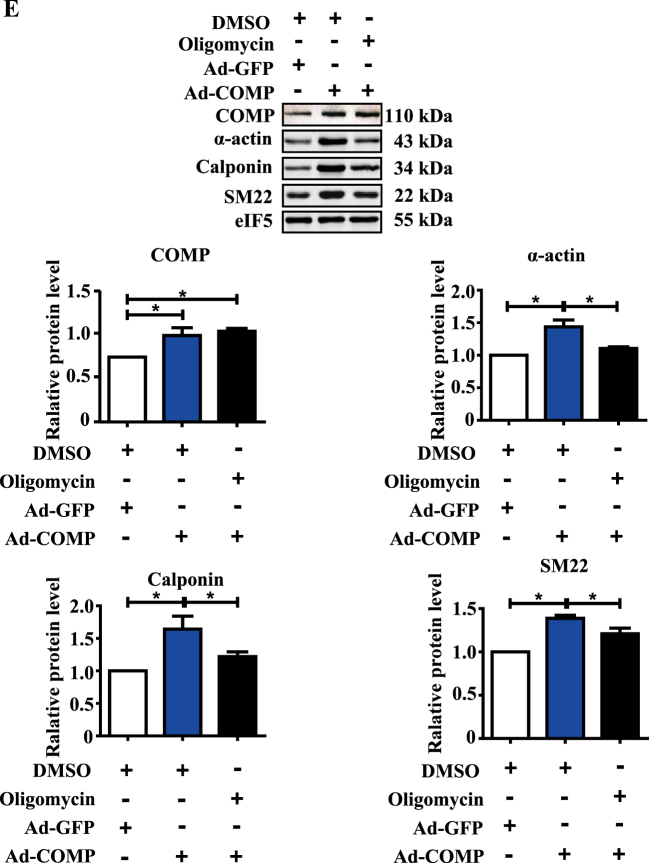
Mitochondrial bioenergetics defects by COMP deficiency contributes to VSMC dedifferentiation. **a** Traces and quantification of the mitochondrial oxygen consumption rates of serum-starved VSMCs stimulated with PGDF-BB (25 µg/L) for 48 h. The data were analyzed using unpaired two-tailed Student’s *t* test and presented as the means ± SD of three independent experiments. *P < 0.05. **b** Traces and quantification of the mitochondrial oxygen consumption rates of VSMCs stimulated with TGF-β (2.5 µg/L) for 48 h. The data were analyzed using unpaired two-tailed Student’s *t* test and presented as the means ± SD of four independent experiments. *P < 0.05. **c** Western blot analysis and quantification of the protein levels of α-actin, calponin and SM22 in VSMC cell lysates upon oligomycin (1 µM, 2 µM) stimulation. The data was analyzed using one-way ANOVA followed by the Student–Newman–Keuls test for post-hoc comparison and presented as the means ± SD of six independent experiments. *P < 0.05. **d** Western blot analysis and quantification of the protein levels of α-actin, calponin and SM22 in VSMC cell lysates upon transfection of vector or PGC1-α plasmid. The data were analyzed using paired two-tailed Student’s *t* test and presented as the means ± SD of three independent experiments. *P < 0.05. **e** Western blot analysis and quantification of the protein levels of COMP, α-actin, calponin and SM22 in VSMC cell lysates infected with COMP adenovirus or COMP adenovirus plus addition of oligomycin (2 µM). The data was analyzed using one-way ANOVA followed by the Student–Newman–Keuls test for post-hoc comparison and presented as the means ± SD of three independent experiments. *P < 0.05

Although accumulating studies have suggested the altered mitochondrial metabolism among different differentiation state of aortic SMCs, there is no direct evidence showing the cause-effect relationship^12^. We first examined the cell energy phenotype and mitochondrial respiration rate of VSMCs under different differentiation states upon PDGF-BB or TGF-β stimulation. PDGF-BB-induced VSMC dedifferentiation paralleled with decreased respiration, whereas the TGF-β-driven contractile phenotype of VSMCs exhibited enhanced mitochondrial respiration (Fig. 5a, b, Supplemental Figure III). We next asked whether the interruption of the oxidative phosphorylation affects the phenotype of VSMCs. Oligomycin is a well-known inhibitor of mitochondrial ATP synthase and oxidative phosphorylation^40^. Interestingly, our data showed oligomycin could cause contractile VSMC dedifferentiation upon serum starvation (Fig. 5c). PGC-1α, a master regulator of mitochondrial biogenesis, upregulates mitochondrial oxidative phosphorylation-related genes and inhibits neointima formation after vascular injury in rats^41^. Similarly, western blotting showed that PGC-1α promoted contractile phenotype of VSMCs (Fig. 5d).

We then asked whether COMP regulates the VSMC phenotype via modulation of mitochondrial respiration. Intriguingly, COMP overexpression by adenovirus no longer maintained the contractile phenotype of VSMCs when mitochondrial respiration was inhibited by oligomycin (Fig. 5e). Thus, COMP-regulated VSMC phenotype, at least in part, is attributed to mitochondrial respiration.

### Mitochondrial proteomics identifies prohibitin 2 as a binding partner of COMP

**Fig. 6:**
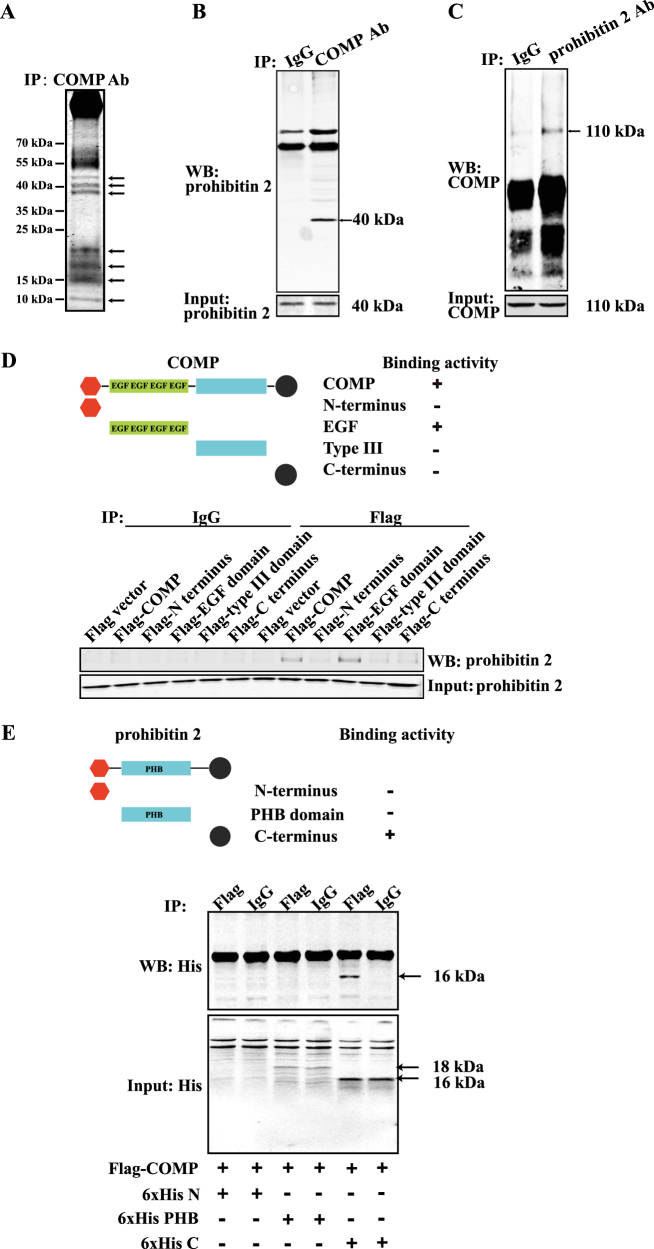
Mitochondrial proteomics identifies prohibitin 2 as a binding partner of COMP. **a** Coomassie brilliant blue-stained gel showing bound proteins resolved by SDS-PAGE. The arrow indicates the bands that were excised and sequenced by mass spectrometry. **b** Co-immunoprecipitation assay of COMP and prohibitin 2 in rat VSMCs. The lysates were immunoprecipitated with anti-prohibitin 2 antibody, and the precipitates were analyzed by immunoblotting with COMP antibody. **c** Co-immunoprecipitation assay of COMP and prohibitin 2 in rat VSMCs. The lysates were immunoprecipitated with COMP antibody, and the precipitates were analyzed by immunoblotting with anti-prohibitin 2 antibody. **d** COS-7 cells were co-transfected with the pcDNA3.1-prohibitin 2 plasmid and flag-COMP plasmid, flag-COMP N-terminal plasmid, flag-COMP EGF domain plasmid, flag-COMP type-3 plasmid, or flag-COMP C-terminal plasmid. Proteins from cells were immunoprecipitated with flag antibody, and the precipitates were analyzed by immunoblotting with anti-prohibitin 2 antibody. **e** HEK293A cells were co-transfected with the Flag-COMP plasmid and 6 × His-prohibitin 2 N-terminal plasmid, 6 × His-prohibitin 2 PHB domain plasmid, or 6 × His-prohibitin 2 C-terminal plasmid. Proteins from cells were immunoprecipitated with the Flag antibody, and the precipitates were analyzed by immunoblotting with the His antibody

To further explore the mechanism underlying COMP regulation on mitochondrial function, we performed mitochondrial proteomics using COMP adenovirus-infected VSMCs. Seven protein bands that specifically immunoprecipitated by COMP antibody but not control IgG were excised and subjected to LC-MS/MS analysis (Fig. 6a). Thus, 602 proteins were identified. Through further DAVID Database analysis, 19 known mitochondria-resident proteins were selected as potential COMP-binding proteins in the mitochondria of VSMCs (Supplemental Table I). Among these proteins, we screened the top-two potential COMP-interacting proteins which have been reported to be involved in the regulation of oxidative phosphorylation (ATP synthase subunit β and prohibitin 2). We initially detected ATP synthase subunit β, the key catalytic subunit of mitochondrial F_1_Fo-ATP synthase, for ATP synthesis^42^. However, ATP synthase subunit β did not co-immunoprecipitate with COMP in VSMCs whereas ATP synthase subunit β was detected in mitochondrial (Supplemental Figure IVA). Next, we tested the potential interaction between COMP and prohibitin 2. The loss of prohibitin 2 impairs respiratory supercomplex formation and causes defects in the mitochondrial respiratory capacity, membrane potential and mitochondrial morphology^43,44^. Through co-immunoprecipitation analysis of the mitochondrial fraction of VSMCs, prohibitin 2 was immunoprecipitated by anti-COMP antibody but not by control IgG (Fig. 6b). Notably, COMP was also exclusively immunoprecipitated by anti-prohibitin 2 antibody (Fig. 6c). In accordance, COMP and prohibitin 2 colocalized in VSMCs as observed under immunofluorescence confocal microscopy (Supplemental Figure IVB). Since prohibitin 1 and prohibitin 2 could form a complex to regulate mitochondrial function^45,46^, we also studied the possible interaction of prohibitin 1 and COMP and found that COMP also immunoprecipitated with prohibitin 1 (Supplemental Figure IVC). To further verify the interaction and identify the specific binding motif of COMP, we subcloned full-length COMP and various domains of COMP (i.e., the N-terminus [N; aa 1–83], epidermal growth factor (EGF) repeat domain [EGF; aa 84–261], type III repeat domain [type III; aa 266–520], and C-terminus [C; aa 521–755]) into the flag-CMV plasmids. COS-7 cells were transfected with the pcDNA3.1-prohibitin 2 plasmid and flag-CMV plasmids that flanked the COMP full length/fragments individually, followed by co-immunoprecipitation using anti-flag antibodies. Both the flag-COMP and flag-EGF domain, but not the other domains, specifically immunoprecipitated prohibitin 2 via flag antibodies but not by IgG (Fig. 6d). Next, we subcloned different domains of prohibitin 2 (i.e., the N-terminus [N; aa 1–39], prohibitin domain [PHB; aa 34–264], and C-terminus [C; aa 201–299]) into the 6 × His-pcDNA3.1 plasmid. HEK293A cells were transfected with full-length flag-COMP and the 6 × His-pcDNA3.1 plasmid that flanked the prohibitin 2 fragments individually, followed by co-immunoprecipitation using anti-Flag antibody. Flag-COMP specifically immunoprecipitated the 6 × His-C terminal domain of prohibitin 2 via Flag antibody but not via the control IgG (Fig. 6e). Taken together, the data revealed that prohibitin 2 directly interacts with COMP at the EGF domain via its C-terminus.

### COMP–prohibitin 2 interaction maintains mitochondrial homeostasis and the VSMC contractile phenotype

**Fig. 7:**
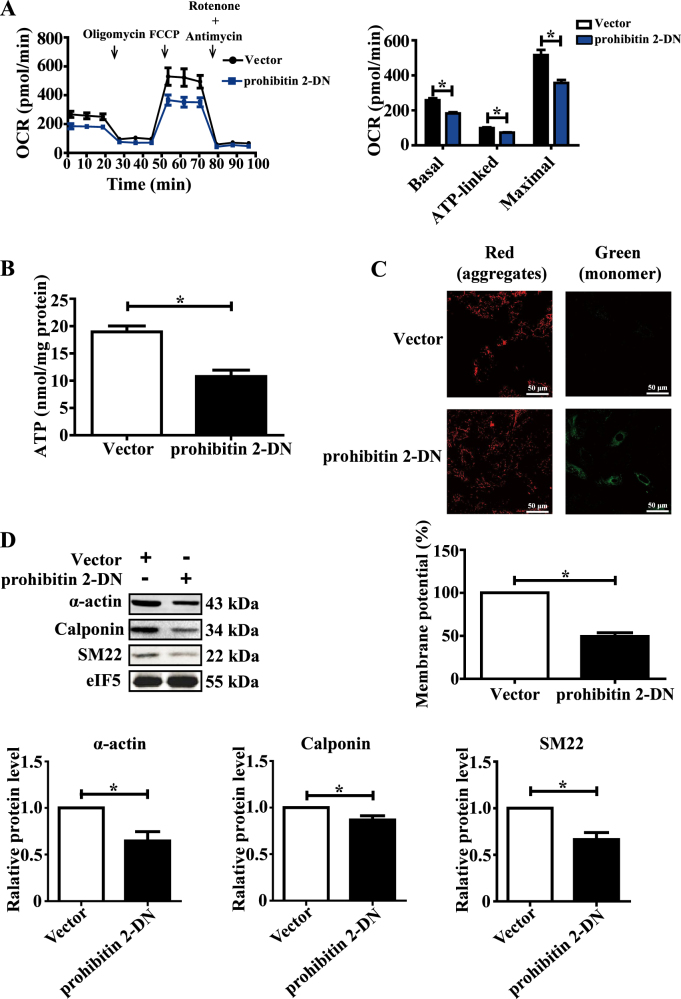

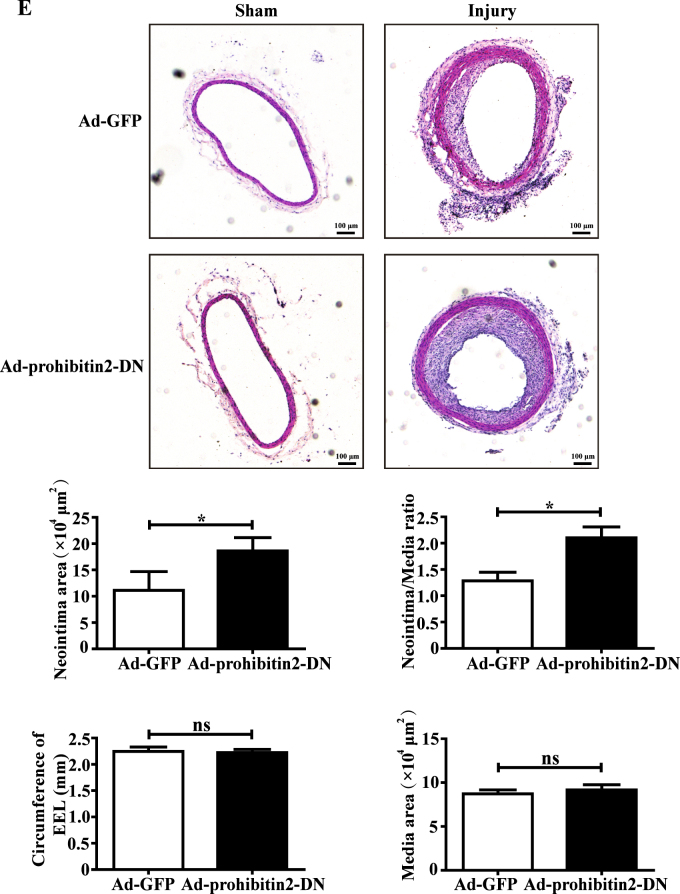
The COMP–prohibitin 2 interaction maintains mitochondrial homeostasis and the VSMC contractile phenotype. **a** VSMCs were transfected with vector or the prohibitin 2-DN plasmid. Quantification of the OCRs. The data was analyzed using paired two-tailed Student’s *t* test and presented as the means ± SD of five independent experiments. *P < 0.05. **b** Intracellular ATP production of rat VSMCs transfected with vector or the prohibitin 2-DN plasmid. The data were analyzed using unpaired two-tailed Student’s *t* test and presented as the means ± SD of three independent experiments. *P < 0.05. **c** JC-1 staining of rat VSMCs transfected with vector or the prohibitin 2-DN plasmid. Scale bar = 50 μm. Quantification was calculated as a quotient of the fluorescence intensities of the red and green fluorescent signals. The data were analyzed using paired two-tailed Student’s *t* test and presented as the means ± SD of three independent experiments. *P < 0.05. **d** Western blot analysis and quantification of the protein levels of α-actin, calponin and SM22 in cell lysates from vector or prohibitin 2-DN plasmid-transfected VSMCs. The data were analyzed using paired two-tailed Student’s *t*-test and presented as the means ± SD of three independent experiments. *P < 0.05. **e** Upper: Representative cross sections of hematoxylin & eosin-stained sham operated and ballooned-injured carotid arteries infected with Ad-GFP or Ad-prohibitin 2-DN at day 14, scale bar = 100 µm. Lower: Quantitative analysis of intima area, ratio of intima to media, circumference of external elastic lamina (EEL) and media area in histological sections from ballooned-injured carotid arteries infected with Ad-GFP or Ad-prohibitin 2-DN at day 14. The data were analyzed using unpaired two-tailed Student’s *t* test and presented as the means ± SD. *N* = 6–8 in each group. *P < 0.05. ns no significance

To further explore the COMP–prohibitin 2 interaction in the modulation of mitochondrial respiration and VSMC differentiation, the plasmid encoding the C-terminus of prohibitin 2 was transfected into VSMCs as a dominant-negative (DN) domain to block the binding between COMP and prohibitin 2 (Supplemental Figure VA). Consequently, transfection of the DN domain into contractile VSMCs markedly inhibited mitochondrial respiration, ATP production, and the membrane potential, greatly mimicking COMP-deficient VSMCs. Meanwhile, VSMCs underwent phenotype transition toward dedifferentiation (Fig. 7a–d). In addition, adenovirus-mediated overexpression of the DN domain in carotid arteries significantly aggravated balloon injury-induced neointima formation (11.14 ± 1.19 vs. 18.62 ± 0.96 × 10^4^ µm^2^) in rats (Fig. 7e and Supplemental Figure VB), indicating that disruption of the COMP–prohibitin 2 interaction led to VSMC dedifferentiation and intimal hyperplasia in vivo. Thus, the COMP–prohibitin 2 interaction plays an essential role in maintaining mitochondrial homeostasis and the VSMC contractile phenotype.

## Discussion

Understanding the molecular mechanisms regulating SMC phenotype transition is critical to elucidating a novel therapeutics for cardiovascular diseases. In the current study, we revealed a pivotal role of the intracellular COMP–prohibitin 2 interaction in maintaining mitochondrial homeostasis and the contractile phenotype of VSMCs. Our data particularly highlight the importance of mitochondrial respiration regulated by COMP–prohibitin 2 in VSMC phenotypic modulation and may shed light on the development of a novel therapeutic strategy.

Mitochondria not only are the powerhouses for ATP production but also play critical roles in ROS generation, inflammation, metabolism and apoptosis^47,48^. Accumulating evidence has linked vascular diseases such as atherosclerosis and pulmonary hypertension to increased mitochondria-generated ROS, accumulation of mitochondrial DNA (mtDNA) damage and progressive respiratory chain dysfunction^49,50^. However, the molecular link of mitochondrial dysfunction to vascular diseases is not fully understood. We previously identified COMP as an important modulator in maintaining vascular homeostasis. COMP^−/−^ mice are more susceptible to post-injury restenosis, atherosclerosis, vascular calcification and thrombosis formation^20,21,51,52^. COMP-degrading proteinase ADAMTS-7 has been demonstrated to be involved in the pathogenesis of atherosclerosis by both human genome-wide association studies and in vivo mouse models^31,53^. Herein, we revealed an unexpected intracellular distribution of COMP and its undisputable role in the maintenance of mitochondrial homeostasis in VSMCs. COMP deficiency led to impaired mitochondrial respiration and ATP production and systemic mitochondrial dysfunction, which may also be involved in the aberrant vascular phenotype in COMP^−/−^ mice.

Of interest, our proteomics analysis identified a mitochondrial binding partner of COMP, prohibitin 2. COMP deficiency shares similar identities with prohibitin 2 deficiency in mitochondrial dysfunction, as previous studies revealed that lack of prohibitin 2 impaired oxidative phosphorylation, reduced mtDNA copy number and attenuated mitochondrial membrane potential^25,54^. In addition, Prohibitin 2 deficiency inhibits the activities of complex I, II and IV in mitochondrial respiratory chain^25^. Although the mRNA levels of mitochondrial respiration complex subunits (ATP5b, Cox5a, Ndufa5 etc.) were significantly dowregulated by COMP deficiency, whether COMP regulates mitochondrial respiration complex activity needs to be further studied. Prohibitin 2 is also associated with many critical mitochondrial proteins, including components of the mitochondrial respiratory chain, mitochondrial structure and cristae formation, mitochondrial transporters, membrane translocases, mitochondrial-mediated translation, and mitochondrial apoptosis^55,56^. A recent study also revealed prohibitin 2 as an inner membrane mitophagy receptor^57^. In the current study, we found that COMP and prohibitin 2 are functionally interdependent in the regulation of mitochondrial respiration via direct interaction. Whether the COMP–prohibitin 2 interaction affects other mitochondrial structures and functions needs to be further explored. In addition, prohibitin 2 is ubiquitously expressed in many other tissues, such as the heart^58^. The mitochondrial COMP–prohibitin 2 interaction may not be limited to VSMCs. We did observe COMP expressed in the mitochondria in cardiomyocytes (data not shown), and COMP deficiency caused spontaneous dilated cardiomyopathy^59^. Whether the COMP–prohibitin 2 interaction in the heart plays essential roles in mitochondrial function and heart failure requires further investigation.

Another interesting finding of our study is that mitochondrial bioenergetics regulated by COMP–prohibitin 2 directs VSMC phenotypic transition. Until recently, little has been known regarding mitochondrial respiration in terms of VSMC function. Interestingly, direct measurement of the cellular bioenergetics of human coronary artery smooth muscle revealed that VSMCs appear to be very similar to cardiac and skeletal muscle cells in terms of oxidative phosphorylation capacity, although VSMCs have a lower mitochondrial content^11^. Aging cells or PDGF-BB-treated dedifferentiated VSMCs have lower oxidative phosphorylation and reduced reserve capacity^18^. Moreover, a metabolic shift from oxidative phosphorylation to glycolysis and substrate selectivity between glucose and fat oxidation were observed in synthetic VSMCs^12,60^. Recent research reported lactate could promote synthetic phenotype in VSMCs^61^.This process was similar to the Warburg effect present in cancer cells that is known to favor glycolysis even when well oxygenated. Our study not only provides further direct evidence that the manipulation of mitochondrial respiration led to VSMC phenotypic transition but also reveals maintaining mitochondrial respiration may inhibit VSMC phenotype switching and alleviate vascular diseases. However, how the alteration of mitochondrial metabolism contributes to the VSMC phenotypic transition needs further investigation.

A surprising finding in this study is the localization of the ECM protein COMP in the mitochondria. COMP belongs to the thrombospondins (TSP) family and is also referred to as TSP-5. Accumulating evidence has indicated that COMP, as an extracellular protein, exerts its functions in both the musculoskeletal and cardiovascular systems by binding to other ECM proteins (e.g., matrilin, fibronectin, and collagen), mitogens (e.g., TGF-β and BMP-2), membrane proteins (e.g., integrin β1 and β3) or thrombin^21,51,52,59,62–64^. To the best of our knowledge, this is the first study to demonstrate that COMP intracellularly resides in an organelle and plays a role in mitochondrial function under physiological conditions. Previously, mutations in the human COMP gene have been linked to the development of two inherited chondrodysplasia and osteoarthritic phenotype, pseudoachondroplasia and multiple epithelial dysplasia, because of aberrant COMP retention in the ER and subsequent ER stress^62^. Of interest, TSP-4, which is also a thrombospondin family member, has been reported as an intracellular adaptive ER stress response effector via binding with the ER luminal domain of activating transcription factor 6a (ATF6a)^65^. More intriguingly, MMP-12, an extracellular matrix metalloproteinase, is transported into virus-infected cells and further translocated into the nucleus, where it acts as a transcription factor^66^. The notion that the same protein with different subcellular localizations plays various roles in cell behavior highlights the complexities of the ECM superfamily proteins.

Our previous study showed the COMP is unregulated by serum starving or TGF-β and repressed by PDGF-BB or serum supplement in VSMCs during phenotype switching^20^. Moreover, we also identified COMP degradation by metalloproteinase ADAMTS-7 during vascular injury or calcification^31,67^. How mitochondrial COMP level was regulated needs further exploration.

Thus, COMP deficiency aggravates VSMC phenotypic switching, at least in part, by disrupting mitochondrial homeostasis maintained by the interaction between COMP and prohibitin 2. Increasing endogenous COMP or improving mitochondrial respiration may help maintain the VSMC contractile phenotype to inhibit several cardiovascular diseases.

## Electronic supplementary material

Supplemental material

Supplementary figure legends
