# Supplementary material for: COMP-prohibitin 2 interaction maintains mitochondrial homeostasis and controls smooth muscle cell identity
Source: Cell Death Dis. 2018 Jun 4;9(6):676. doi: 10.1038/s41419-018-0703-x (PMC5986769; doi:10.1038/s41419-018-0703-x)
Supplement: Supplementary file 1 — Supplemental material [file 41419_2018_703_MOESM1_ESM.docx]

**SUPPLEMENTAL MATERIAL**

**Detailed Attribution of Authorship**

Yiting Jia, Meili Wang, Yi Fu and Wei Kong designed experiments and helped write the manuscript.

Chenfeng Mao, Fang Yu, Yingbao Wang and Rui Xiao contributes to part of the experiments.

Changtao Jiang, Lemin Zheng, Qingbo Xu, Ming Zheng make suggestions to this project.

In Figure 1, Meili Wang generated the data, Yiting Jia prepared panel A, B and C. Yiting Jia generated the proteomics data and prepared panel D and E.

In Figure 2, Yiting Jia and Rui Xiao generated the data and assembled the figure.

In Figure 3, Meili Wang generated the data and prepared panel A-D. Yiting Jia generated the data and prepared panel E-G.

In Figure 4, Meili Wang generated the data and prepared panel A, C and D. Yiting Jia generated the data and prepared panel B.

In Figure 5, Yiting Jia and Chengfeng Mao generated the data and assembled the figure.

In Figure 6, Meili Wang generated the data and prepared panel A-D. Yiting Jia generated the proteomics data and prepared panel E.

In Figure 7, Yiting Jia generated the data and prepared panel A-D. Yiting Jia and Yingbao Wang generated the data and prepared panel E.

**Supplemental Methods**

**Cell Culture**

Rat smooth-muscle embryonic thoracic aorta cell line A7r5, human aortic smooth muscle cell line (T/G HA VSMCs), HEK293A cells and COS-7 cells were purchased from ATCC and cultured in high-glucose Dulbecco’s modified Eagle’s medium (DMEM) with 10% FBS. Primary aortic VSMCs were isolated from the aortas of 8-week-old mice. Following mouse euthanasia, aortas were harvested and cut into small pieces. To remove the endothelial cells, these pieces were digested with 1 mg/ml trypsin (HyClone) at 37 °C for 10 min. After centrifugation, the precipitate was resuspended with 10 mg/ml collagenase type I (Gibco) for 6-8 h. Gelatin (0.1%) was pre-laid onto the culture dishes before the cells were seeded. The cell cultures contained > 95% VSMCs as determined by SM22 staining. The cells were cultured in complete DMEM containing 10% fetal bovine serum (FBS, HyClone) and were passaged by 0.25% trypsin digestion. The cells of passages 3-8 were used for experiments.

**Immunofluorescence**

Cells were washed with PBS twice and were fixed with 4% paraformaldehyde for 10 min. The cells were subsequently permeabilized by 0.1% Triton X-100, followed by 30 min of incubation with 3% BSA. For dual immunofluorescence, the cells were incubated with antibodies against COMP (1:50, Abcam), ERp5 (1:100, Abcam) or TGN46 (1:1000, Abcam) followed by incubation with secondary TRITC-conjugated goat anti-rat IgG (1:400) or FITC-conjugated goat anti-rabbit IgG (1:400) (Rockland Inc., Gilbertsville, PA). For mitochondrial staining, the cells were washed with PBS and incubated in Mito-Tracker dye (50 nM) at 37 °C for 30 min. Nuclei were stained with Hoechst 33342. Confocal laser scanning microscopy (Leica, Germany) was applied for fluorescence observation. For cytoskeleton staining, the cells were washed with PBS and fixed with 4% paraformaldehyde for 10 min. The cytoskeleton was stained with phalloidin (1:100, Cell Signaling Technology). Nuclei were stained with Hoechst 33342. Confocal laser scanning microscopy (Leica, Germany) was applied for fluorescence observation.

**RT-qPCR**

Total RNA was extracted from mouse VSMCs, and equal amounts (2 μg) were reverse transcribed into cDNA. The SYBR Green 2× PCR mix (TransGen Biotech, Beijing, China) was used according to the manufacturer’s instructions. RT-qPCR amplification involved the use of an Mx3000 Multiplex Quantitative PCR System (Stratagene Corp, La Jolla, CA, USA) and SYBR Green I reagent with normalization to the internal control β-actin. Sequence of all the primers used were shown in Supplemental Table II.

**Transfection of Small Interfering RNA**

Small interfering RNA (siRNA) was purchased from GenePharma Co., Ltd. (Shanghai). The sequences that corresponded to the siRNA of rat COMP were sense, 5’-AGAAACUUGAGCUGUGUUGAUGCC-3’, and anti-sense, 5’-GGCUAUCAAGACAGCUCAAGUUUCU-3’. A scrambled stealth RNAi duplex served as a negative control. *In vitro* siRNA transfection (20 nM) of rat VSMCs was performed using RNAiMAX (Invitrogen, CA, USA). The transfection procedures followed the manufacturers’ instructions.

**Measurement of Cellular ATP**

Intracellular ATP was measured using the ATP-lite Luminescence Assay kit (Vigorous Biotechnology) following the manufacturer’s protocol. Briefly, cells were washed twice with ice-cold PBS prior to being lysed with lysis buffer and were shaken on the vortex generator for 30 seconds. Following centrifugation at 12,000 rpm for 1 min, the supernatant was incubated with freshly prepared assay reagent and was subsequently subjected to bioluminescent detection.

**Measurement of the Mitochondrial Membrane Potential**

Cells were incubated with JC-1 fluorescent dye (Invitrogen, CA, USA) at a concentration of 5 g/ml for 20 min and subsequently washed with PBS. The mitochondrial membrane potential was detected by confocal microscopy for observation and flow cytometry for quantification. A red fluorescent JC-1 signal is indicative of healthy cells with a high △Ψm, whereas a green fluorescent JC-1 signal is indicative of unhealthy cells with a low △Ψm.

**qPCR for mtDNA Copy Number**

For the assessment of the rat VSMCs mtDNA copy number, we used a unique mitochondrial gene, ND1, whose primers are as follows: Sense: 5’-ATTCTAGCCACATCAAGTCTTT-3’, Anti-sense: 5’-GGAGGACGGATAAGAGGATAAT-3’. QPCR was performed using 20 ng of sample DNA in 20 μl reactions. β-globin was used as an internal control.

**Proteomic Analysis**

Mitochondria were isolated from rat VSMCs infected with COMP adenovirus for 48 h. The lysates of the mitochondria were incubated with anti-COMP antibody or control IgG. The precipitated proteins were denatured with 2× SDS sample buffer at 95 °C for 5 min and were subsequently loaded onto 6-20% gradient gels. Following Coomassie brilliant blue staining, the protein bands present were exclusively excised. The proteins in each band were further extracted and analyzed by liquid chromatography and tandem mass spectrometry. Mitochondrial protein identification was performed according to the DAVID Functional Annotation Bioinformatics Microarray Analysis (<http://david.abcc.ncifcrf.gov/>).

**Co-immunoprecipitation**

Whole-cell lysates were incubated with 2 μg of anti-COMP or anti-prohibitin 2 antibodies at 4 °C overnight. Next, 20 μl of protein A/G agarose beads (Santa Cruz Biotechnology) were added to the samples and were incubated for 3 h at 4 °C. After the supernatant was removed, the beads were washed six times with cold wash buffer. The precipitated proteins were resolved using 10% SDS-PAGE and were immunoblotted with anti-prohibitin 2 or anti-COMP antibodies, respectively. Normal rabbit IgG antibodies were used as a negative control.

COS-7 cells were co-transfected with Flag-CMV vectors that encoded COMP full length or various fragments (N-terminal, EGF domain, type-3 domain, or C-terminal) and full-length prohibitin 2 for 48 h, respectively. The cell lysates were incubated with anti-Flag antibody and were subsequently immunoblotted with anti-prohibitin 2 antibody.

In another study, HEK293A cells were co-transfected with 6×His vector-encoded prohibitin 2 fragments (N-terminal, PHB domain or C-terminal) and full-length COMP for 48 h, respectively. The cell lysates were incubated with anti-Flag antibody and were subsequently immunoblotted with anti-His antibody.

**Morphometric Analysis of Rat Carotid Arteries**

Rats were euthanatized by CO_2_ inhalation, followed by perfusion with PBS and fixation with 4% paraformaldehyde. The carotid arteries were dissected and further embedded in OCT compound. Cryosections (7 µm thickness, 150 µm apart) were taken from the middle portion of the balloon-injured segment, and 10 serial slices of each sample were analyzed by hematoxylin/eosin staining and Image pro plus software (Media Cybernetics, MD, USA).

**Supplemental Figures and Figure legends**

**
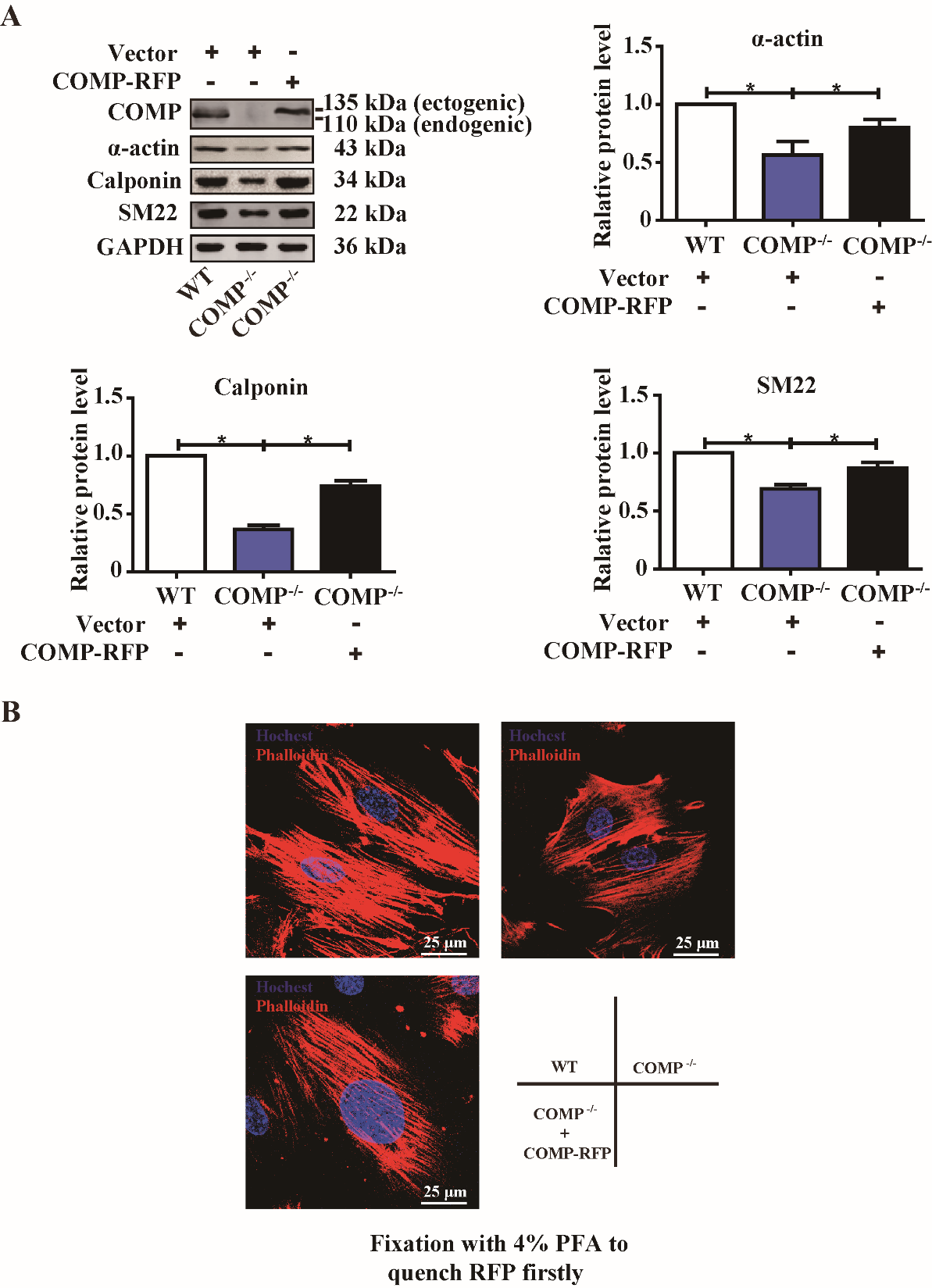
Supplemental Figure I.** **A**, Western blot analysis of the protein levels of COMP (including both endogenic and ectogenic), α-actin, calponin and SM22 in cell lysates from WT, COMP^-/-^ and COMP^-/-^ transfected with COMP-RFP plasmid VSMCs. The data was analyzed using paired two-tailed Student’s *t*-test and presented as the means ± SD of three independent experiments. **P*<0.05. **B**, Phalloidin staining of WT, COMP^-/-^ and COMP^-/-^ VSMCs transfected with COMP-RFP plasmid. Scale bar = 25 μm.


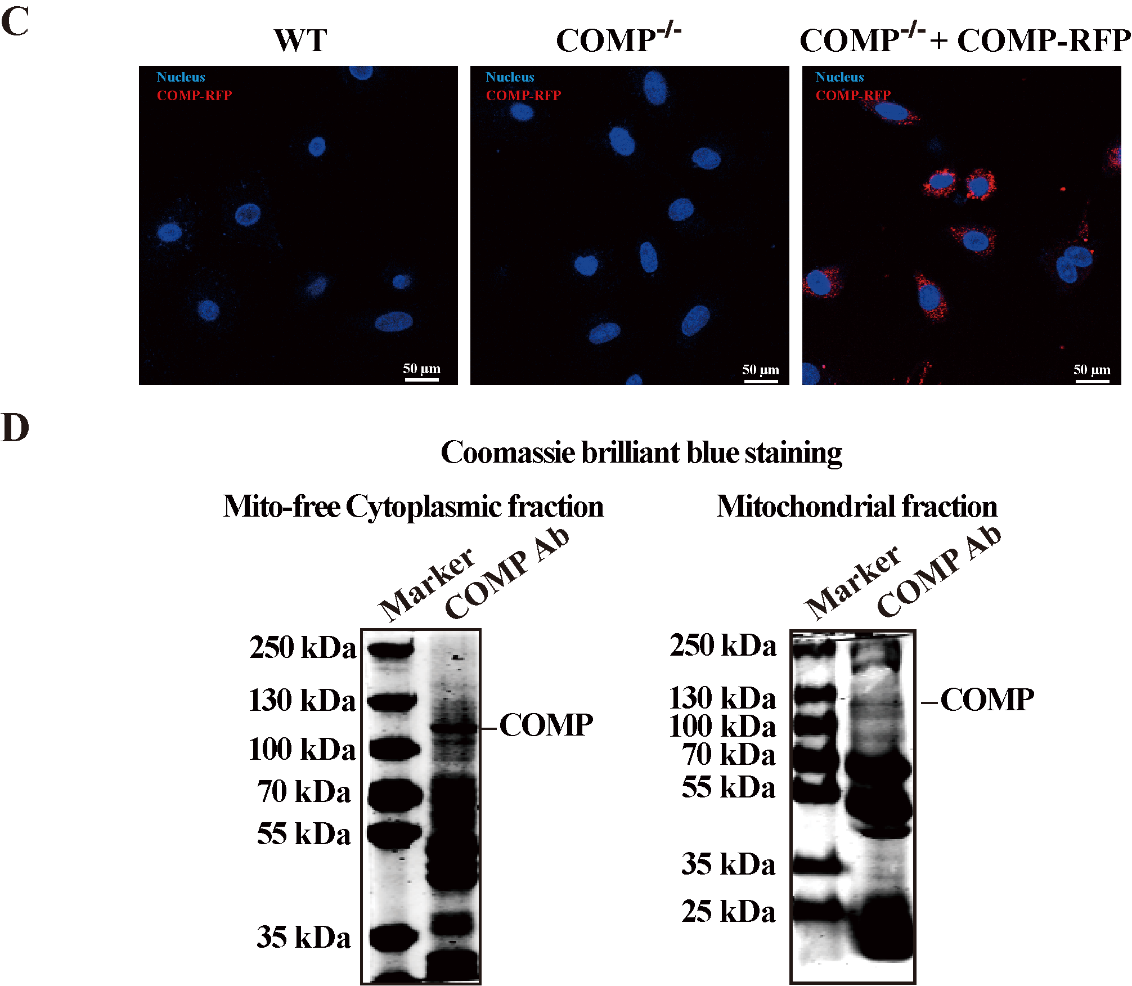


**Supplemental Figure I. C**, Intracellular localization of COMP-RFP in wild-type, COMP^-/-^ and COMP-RFP transfected COMP^-/-^ VSMCs as demonstrated by confocal fluorescence microscopy. Scale bar = 50 μm. **D**, HEK293A cells were transfected with full length COMP plasmid, followed by non-mitochondrial cytoplasm and mitochondria separation and then immunoprecipitation using anti-COMP antibody. After Coomassie brilliant blue staining, the protein band of COMP (110 kDa) was excised, and the N-terminus was labeled with a dimethyl moiety, followed by subjection to LC-MS/MS analysis.


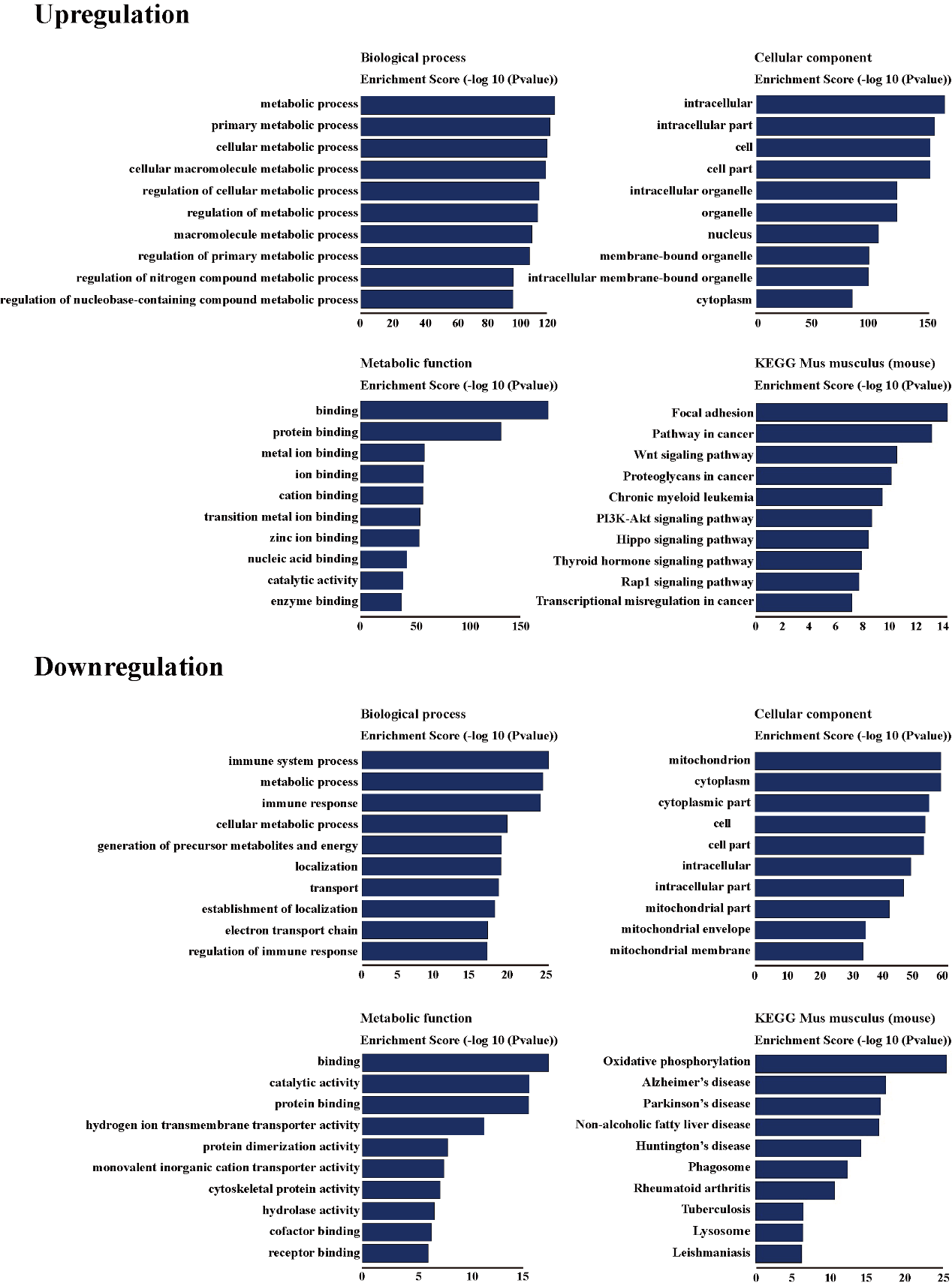


**Supplemental Figure II.** Microarray analysis of GO pathways, including biological processes, cellular components and metabolic function and KEGG pathways, upregulated or downregulated by COMP deficiency.

**
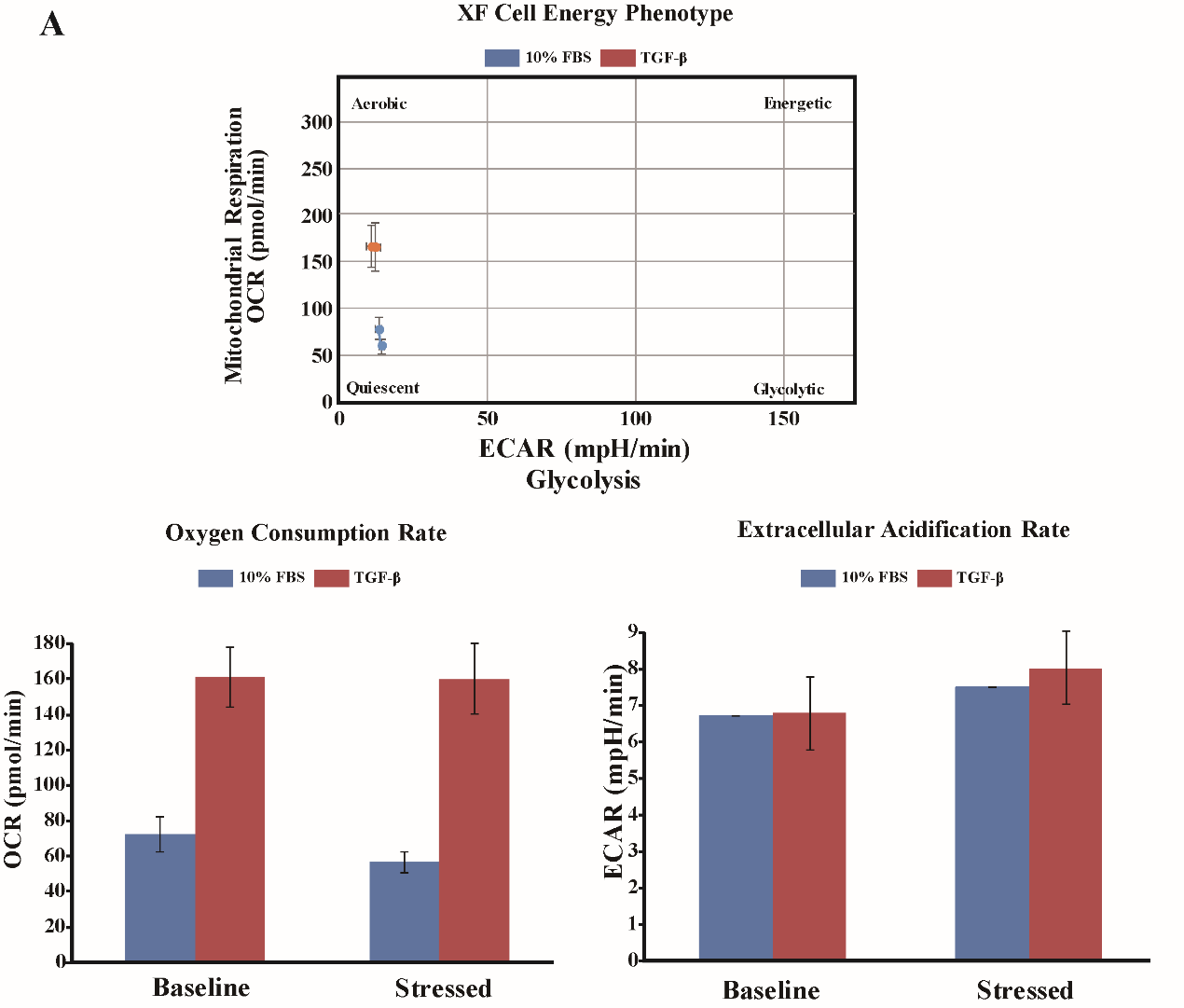
 Supplemental Figure III.** **A**, Cell energy phenotype assay of VSMCs stimulated with TGF-β (2.5µg/L) for 48 hours.


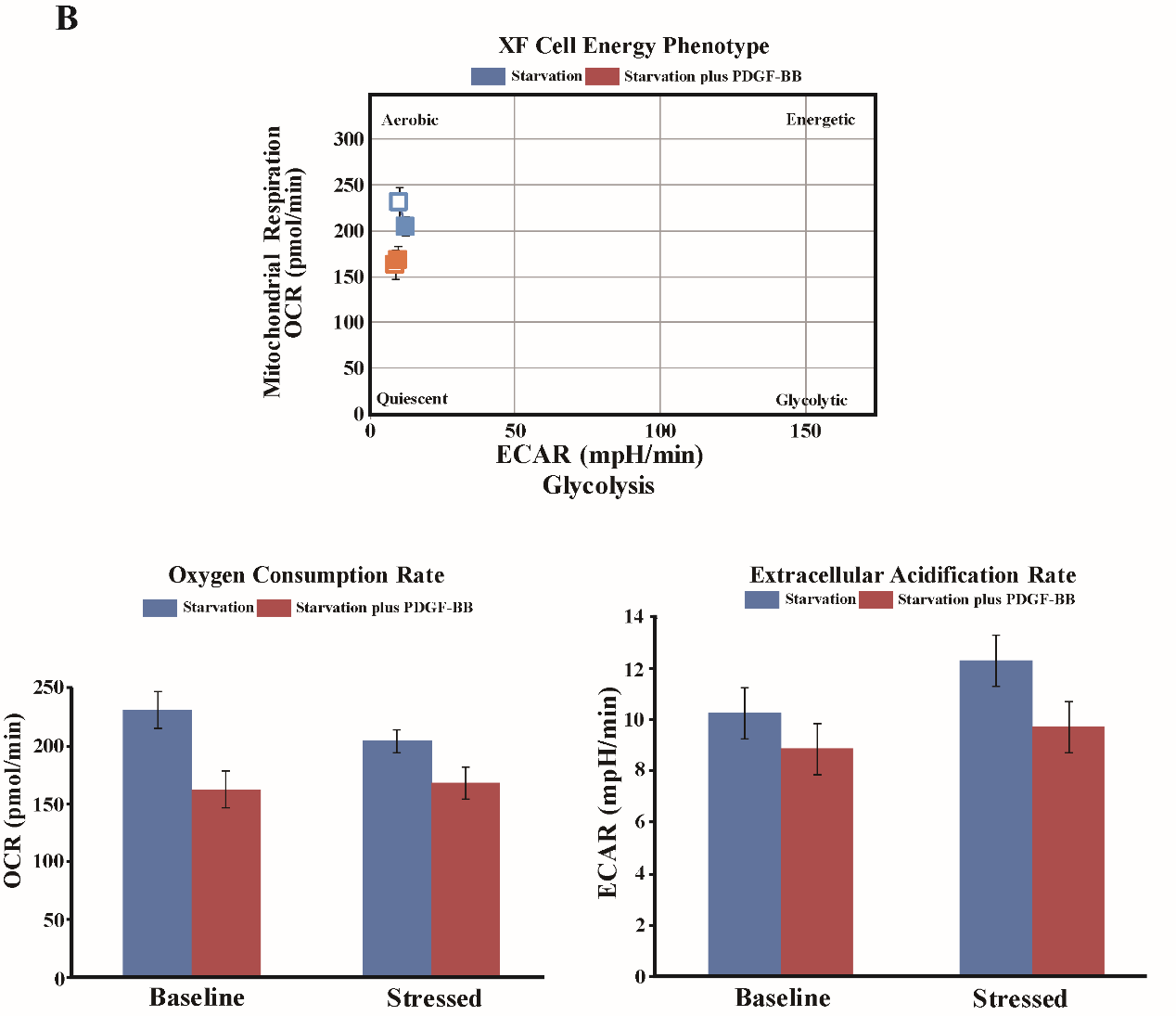


**Supplemental Figure III.** **B**, Cell energy phenotype assay of serum-starved VSMCs stimulated with PGDF-BB (25 µg/L) for 48 hours.

**
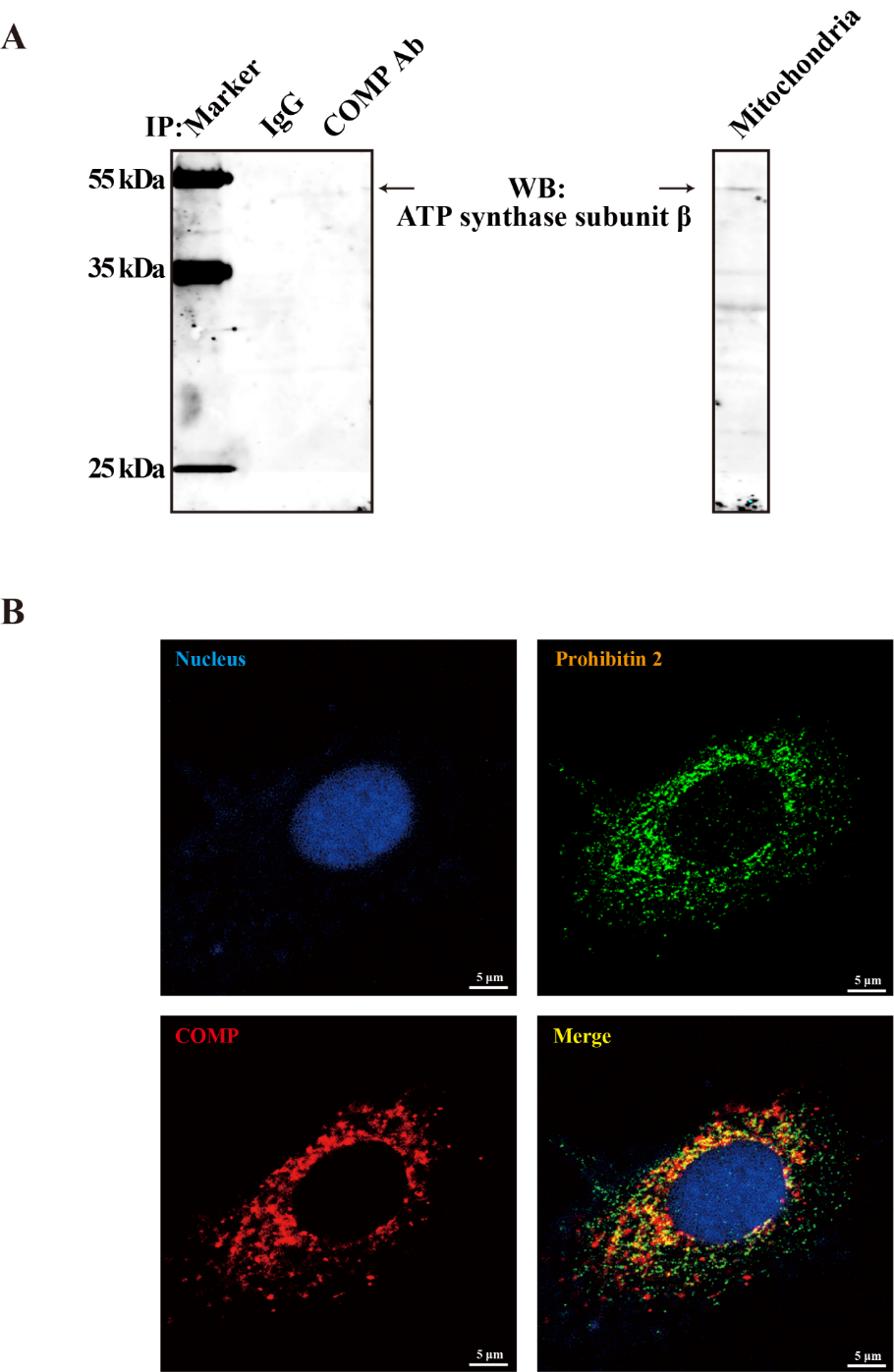
**

**Supplemental Figure IV.** **A**, Co-immunoprecipitation assay of COMP and ATP synthase subunit β in mitochondria from rat VSMCs. Lysates were immunoprecipitated with COMP antibody, and the precipitates were analyzed by immunoblotting with the ATP synthase subunit β antibody. Rabbit IgG was used as a negative control for IP. Mitochondrial proteins were used as a positive control to indicate ATP synthase subunit β. **B**, Colocalization of COMP and prohibitin 2 as indicated by confocal fluorescence microscopy. Scale bar = 5 μm.


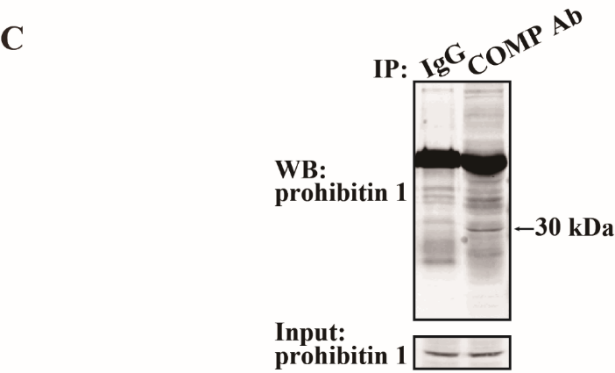


**Supplemental Figure IV.** **C**, Co-immunoprecipitation assay of COMP and prohibitin 1 in mitochondria from rat VSMCs. Lysates were immunoprecipitated with anti-COMP antibody, and the precipitates were analyzed by immunoblotting with prohibitin 1 antibody. Rabbit IgG was used as a negative control for IP.


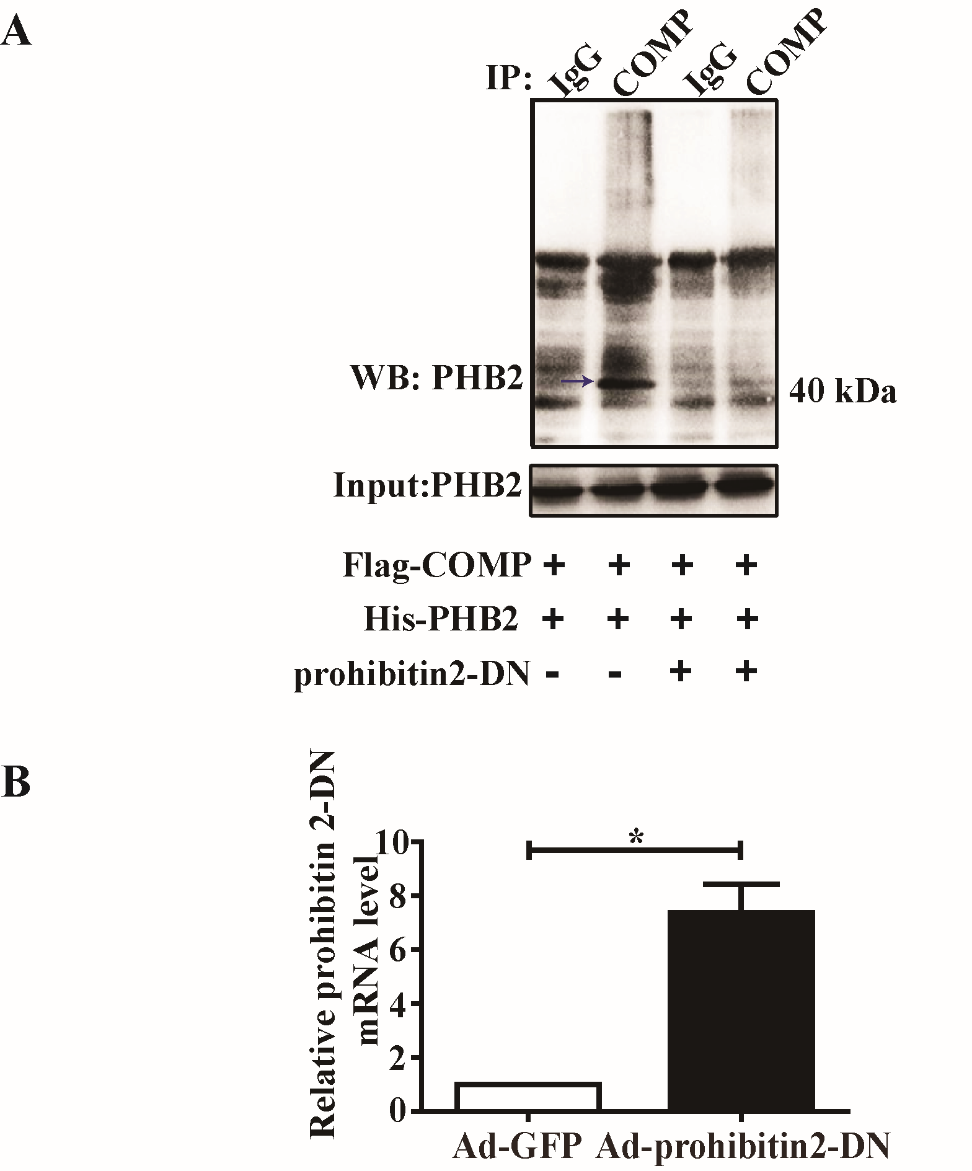


**Supplemental Figure V.** **A**, HEK293A cells were treated with the Flag-COMP plasmid and 6×His-prohibitin 2 plasmid with or without the prohibitin 2-DN plasmid. Proteins from cells were immunoprecipitated with the COMP antibody, and the precipitates were analyzed by immunoblotting with the prohibitin 2 antibody. Rabbit IgG was used as a negative control for IP. **B**, RT-qPCR validation of prohibitin 2-DN fragment expression in ballooned-injured carotid arteries infected with Ad-GFP or Ad-prohibitin 2-DN at day 4. The data was analyzed using paired two-tailed Student’s *t*-test and presented as the means ± SD. N=3 in each group. **P*<0.05.

**Supplemental Table I. LC-MS/MS analysis of mitochondrial proteins co-immunoprecipitated with the COMP antibody.**


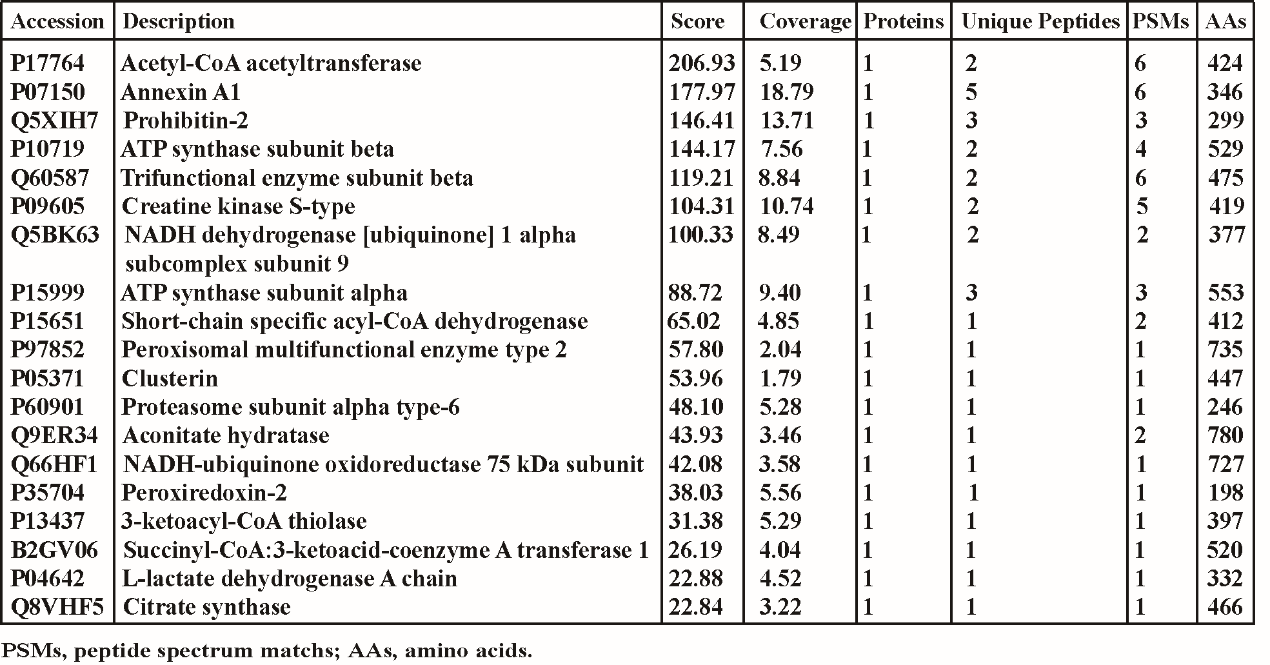


**Supplemental Table II. Sequence of the primers used for RT-qPCR.**

**
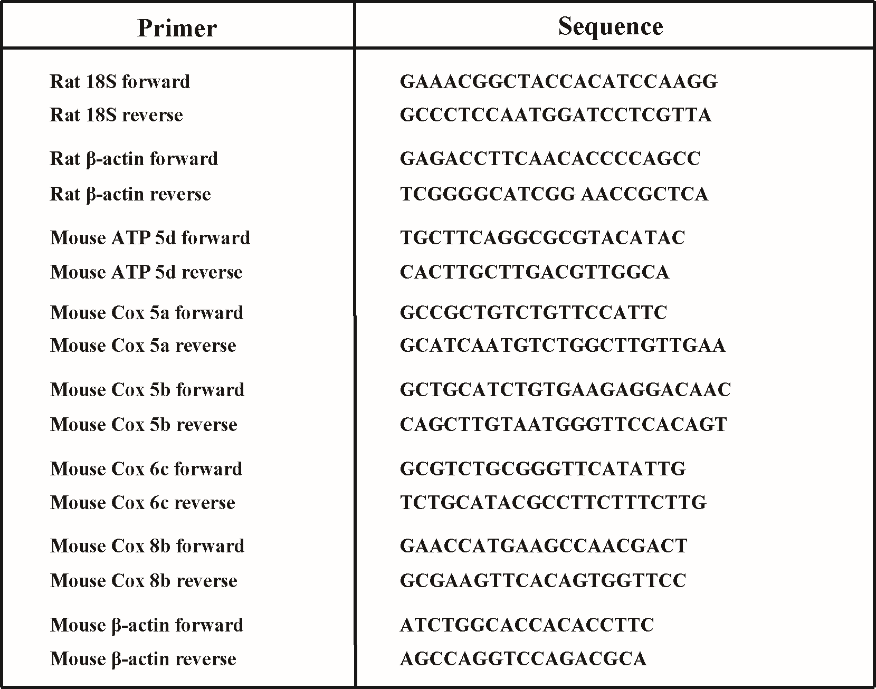
**

**Supplemental Table III. Sequence of the primers used for subcloning.**

**
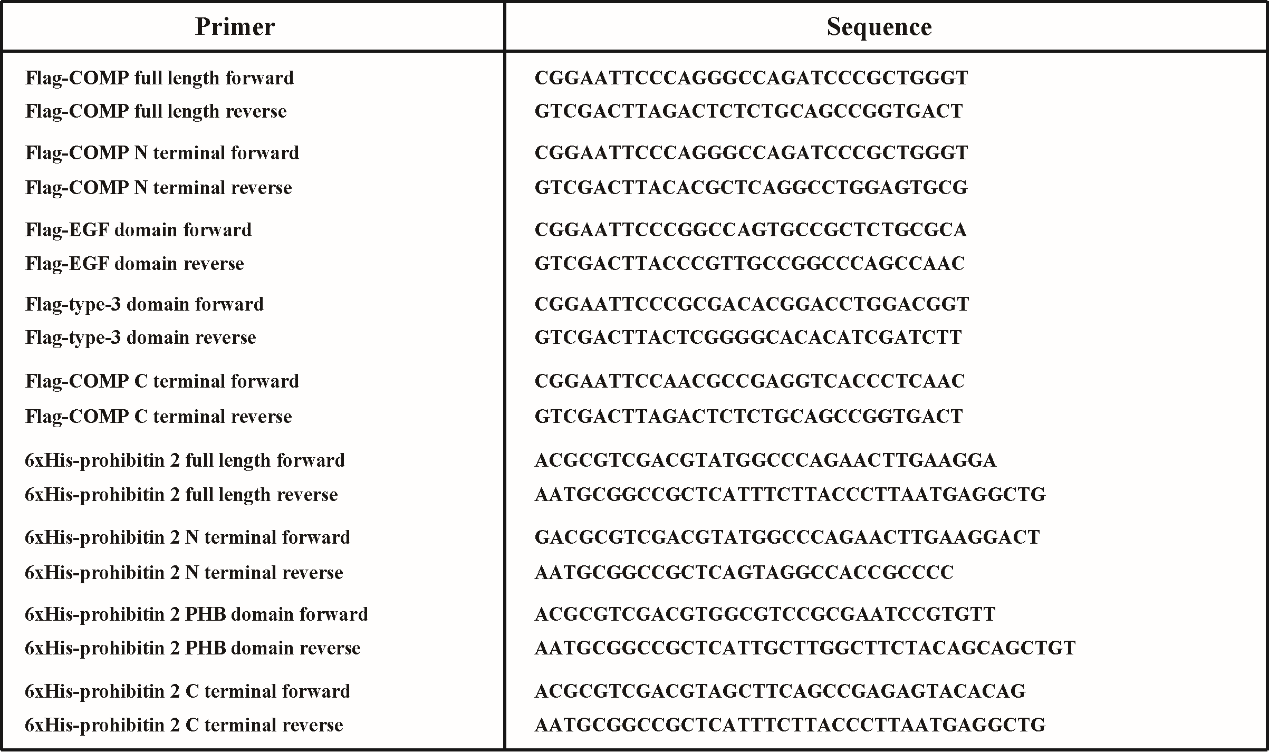
**
