# Supplementary material for: COMP-prohibitin 2 interaction maintains mitochondrial homeostasis and controls smooth muscle cell identity
Source: Cell Death Dis. 2018 Jun 4;9(6):676. doi: 10.1038/s41419-018-0703-x (PMC5986769; doi:10.1038/s41419-018-0703-x)
Supplement: Supplementary file 2 — Supplementary figure legends [file 41419_2018_703_MOESM2_ESM.docx]

**Supplementary Figure Legends**

**Supplemental Figure I.** **A**, Western blot analysis of the protein levels of COMP (including both endogenic and ectogenic), α-actin, calponin and SM22 in cell lysates from WT, COMP^-/-^ and COMP^-/-^ transfected with COMP-RFP plasmid VSMCs. The data was analyzed using paired two-tailed Student’s *t*-test and presented as the means ± SD of three independent experiments. **P*<0.05. **B**, Phalloidin staining of WT, COMP^-/-^ and COMP^-/-^ VSMCs transfected with COMP-RFP plasmid. Scale bar = 25 μm. **C**, Intracellular localization of COMP-RFP in wild-type, COMP^-/-^ and COMP-RFP transfected COMP^-/-^ VSMCs as demonstrated by confocal fluorescence microscopy. Scale bar = 50 μm. **D**, HEK293A cells were transfected with full length COMP plasmid, followed by non-mitochondrial cytoplasm and mitochondria separation and then immunoprecipitation using anti-COMP antibody. After Coomassie brilliant blue staining, the protein band of COMP (110 kDa) was excised, and the N-terminus was labeled with a dimethyl moiety, followed by subjection to LC-MS/MS analysis.

**Supplemental Figure II.** Microarray analysis of GO pathways, including biological processes, cellular components and metabolic function and KEGG pathways, upregulated or downregulated by COMP deficiency.

**Supplemental Figure III.** **A**, Cell energy phenotype assay of VSMCs stimulated with TGF-β (2.5µg/L) for 48 hours. **B**, Cell energy phenotype assay of serum-starved VSMCs stimulated with PGDF-BB (25 µg/L) for 48 hours.

**Supplemental Figure IV.** **A**, Co-immunoprecipitation assay of COMP and ATP synthase subunit β in mitochondria from rat VSMCs. Lysates were immunoprecipitated with COMP antibody, and the precipitates were analyzed by immunoblotting with the ATP synthase subunit β antibody. Rabbit IgG was used as a negative control for IP. Mitochondrial proteins were used as a positive control to indicate ATP synthase subunit β. **B**, Colocalization of COMP and prohibitin 2 as indicated by confocal fluorescence microscopy. Scale bar = 5 μm. **C**, Co-immunoprecipitation assay of COMP and prohibitin 1 in mitochondria from rat VSMCs. Lysates were immunoprecipitated with anti-COMP antibody, and the precipitates were analyzed by immunoblotting with prohibitin 1 antibody. Rabbit IgG was used as a negative control for IP.

**Supplemental Figure V.** **A**, HEK293A cells were treated with the Flag-COMP plasmid and 6×His-prohibitin 2 plasmid with or without the prohibitin 2-DN plasmid. Proteins from cells were immunoprecipitated with the COMP antibody, and the precipitates were analyzed by immunoblotting with the prohibitin 2 antibody. Rabbit IgG was used as a negative control for IP. **B**, RT-qPCR validation of prohibitin 2-DN fragment expression in ballooned-injured carotid arteries infected with Ad-GFP or Ad-prohibitin 2-DN at day 4. The data was analyzed using paired two-tailed Student’s *t*-test and presented as the means ± SD. N=3 in each group. **P*<0.05.
